# Applying fractional calculus to malware spread: A fractal-based approach to threat analysis

**DOI:** 10.1371/journal.pone.0313914

**Published:** 2025-01-08

**Authors:** Nausheen Razi, Muhammad Bilal Riaz, Ambreen Bano, Tayyab Kamran, Umar Ishtiaq, Anum Shafiq

**Affiliations:** 1 Department of Mathematics, Quaid-i-Azam University, Islamabad, Pakistan; 2 IT4Innovations, VSB – Technical University of Ostrava, Ostrava, Czech Republic; 3 Department of Computer Science and Mathematics, Lebanese American University, Byblos, Lebanon; 4 Department of Mathematics and Statistics, Ripha International University, Islamabad, Pakistan; Kwame Nkrumah University of Science and Technology, GHANA

## Abstract

Malware is a common word in modern era. Everyone using computer is aware of it. Some users have to face the problem known as Cyber crimes. Nobody can survive without use of modern technologies based on computer networking. To avoid threat of malware, different companies provide antivirus strategies on a high cost. To prevent the data and keep privacy, companies using computers have to buy these antivirus programs (software). Software varies due to types of malware and is developed on structure of malware with a deep insight on behavior of nodes. We selected a mathematical malware propagation model having variable infection rate. We were interested in examining the impact of memory effects in this dynamical system in the sense of fractal fractional (FF) derivatives. In this paper, theoretical analysis is performed by concepts of fixed point theory. Existence, uniqueness and stability conditions are investigated for FF model. Numerical algorithm based on Lagrange two points interpolation polynomial is formed and simulation is done using Matlab R2016a on the deterministic model. We see the impact of different FF orders using power law kernel. Sensitivity analysis of different parameters such as initial infection rate, variable adjustment to sensitivity of infected nodes, immune rate of antivirus strategies and loss rate of immunity of removed nodes is investigated under FF model and is compared with classical. On investigation, we find that FF model describes the effects of memory on nodes in detail. Antivirus software can be developed considering the effect of FF orders and parameters to reduce persistence and eradication of infection. Small changes cause significant perturbation in infected nodes and malware can be driven into passive mode by understanding its propagation by FF derivatives and may take necessary actions to prevent the disaster caused by cyber crimes.

## 1 Introduction

Malware is a program tailored to impair, interrupt or damage a computer chain or in cyberspace. Hackers use their technical skills to develop such programs for stealing important and personal information. It normally occurs in the form of a malicious code. Different types of malware propagate differently. They may self-propagate, or their propagation may be through user interactions via internet or sharing devices like Wifi/Bluetooth etc. Most known types of malware are: Virus, Worms, Adware, Spyware, Trojan, Rootkit, Backdoors, Keyloggers, Ransomware, Cookies, Sniffers, Botnet, Spam, Mobile malware [1–3]. The software has been developed to deal with such program or malware according to its nature. The software is usually called antivirus. It can detect and kill malware. Moreover, it may be installed in computers to act as shield against propagation of malware [4, 5].

To develop effective antivirus software against some particular malware one should know how the malware propagates and works in computers. For this purpose, Feng et al. [6] presented a model of propagation of malware through internet via three states: susceptible, infected and recovered (removed). They assumed that total numbers of nodes (computers) in a network at time *σ* are *N*(*σ*) along with the assumption that each node changes with respect to time in three states. They described that susceptible state (Δ) represents a node which has a weakness that the malware can easily exploit it, infected state (ℵ) shows that when it is infected it can infects its neighboring nodes and still infectious and recovered/removed state (Θ) represents that a detection tool has been installed that is used to identify and overcome a malware. Considering these definitions of states they represented the model in the form of ODES as:

$$\begin{array}{c} \begin{array}{cc} \frac{d\Delta }{d\sigma } & =\Pi \theta -\beta_{0}f\left(ℵ\left(\sigma \right)\right)\Delta \left(\sigma \right)-\left(\mu +\nu \right)\Delta \left(\sigma \right)+\zeta \Theta \left(\sigma -\tau \right),\\ \frac{dℵ}{d\sigma } & =\beta_{0}f\left(ℵ\left(\sigma \right)\right)\Delta \left(\sigma \right)-\left(\mu +\kappa \right)ℵ\left(\sigma \right),\\ \frac{d\Theta }{d\sigma } & =(1-\Pi )\theta +\nu \Delta (\sigma )+\kappa ℵ(\sigma )-\zeta \Theta (\sigma -\tau )-\mu \Theta (\sigma ), \end{array} \end{array}$$ (1.1)

where Π shows the susceptible rate of new nodes, *θ* shows the number of new nodes, *ζ* is the loss rate of immunity of the recovered nodes, *μ* is the replacement rate, *ν* is the real time immune rate of antivirus strategies, *κ* is the recovered rate of infected nodes, *τ* is the change in time, *β*_0_ is the initial infection rate and $f\left(ℵ\left(\sigma \right)\right)=\frac{ℵ(\sigma )}{1+\alpha ℵ(\sigma )}$ satisfies the assumptions as in [6]. The initial conditions are defined by Δ(0) = Δ_0_ ≥ 0, ℵ(0) = ℵ_0_ ≥ 0 and Θ(0) = Θ_0_ ≥ 0. Moreover, *N*(*σ*) = Δ(*σ*) + ℵ(*σ*) + Θ(*σ*) as given above.

As the time passed, due to the increase in the spread of virus and its different aspects we have to study the mathematical models in fractional calculus instead of classical. Fractional calculus is a stereotype of classical. Its concept is known since 1695 when L’Hospital asked Leibnitz about the fractional power of derivative. By replacing usual operators from classical to the fractional, we get more accurate results in many physical problems [7]. Many definitions of fractional derivatives were found [8–11]. Several fractional derivatives and integrals in extended form as with variable order [12, 13] were defined to solve different forms of differential equations and functions but there was a problem that these operators had singular kernel. To solve this problem, fractional operators were found without singular kernels [14, 15] and in refined form with non local as well [16]. Then more advanced papers were published in which flaws of previous papers were removed [17–19].

The notion of fractional operators had not been much worthy for modeling the complex problems of real world. These complex real world problems can be based on those physical occurrences that show fractal behaviour. To handle this type of problems a nonstandard derivative was introduced [20] known as fractal derivative which scales independent variable. In [20] Atangana introduced novel concept of differentiation. The term fractal- fractional (FF) derivative was used in the paper where two concepts of fractal derivative and fractional derivative were combined. He derived fractal fractional derivatives in Caputo sense and in Riemann-Liouville sense also with three different forms of kernels mentioned above. Atangana presented the new definition of Fractal Laplace transform and then used it to solve the fractal differential equation and found corresponding integral.

Malware constitute a chaotic behaviour in the real world and it is random and unpredictable in a nonlinear rule. Phenomena of malware relates to data heterogeneities that cannot be well-defined using other forms of derivatives [21]. To study the complexity of such chaotic system, fractal fractional derivative is much better than classical one [19, 22, 23]. We find detail in [8, 20, 24–28] for more details. To see the memory effect, earlier fractional derivative of variable order [12, 13] were used but recently fractal fractional orders are used to check memory effect [29]. Rezapour et al. [30] provided a theoretical and numerical analysis of two strain (FF) model of meningitis. Recently, fractal fractional derivatives with its theory and numerical aspects were applied for some most important issues like intervention measures in a mathematical model for monkeypox and COVID-19 co-dynamics [31] and for Ebola [32] and most common problem of our countries “corruption” [33]. Poria and Dhiman [34] examined existence and uniqueness theorem for ODES. Authors in [35–37] worked on stability of different equations in the context of Banach spaces.

Malware is a generic issue and many authors have discussed different mathematical models to explain its extremities. Due to its complex features involving chaotic behavior, heterogeneities and memory effect, some authors tried to solve it using the concept of fractional calculus and in advance form of fractal fractional theory. Till now we have seen the models which have a simple nature. So we decided to investigate a more complex mathematical model as presented by Feng et al. This model has a variable infection rate which gives a deep insight of the behavior of malware. Moreover, infection rate is defined as a nonlinear function of infected nodes. To better understand the behavior of such type of malware and develop antivirus software to overcome the malware, we decided to deal this model by converting it into fractal fractional mathematical model with power law kernel initially. We also tried to find the impacts of different parameters on malware propagation for integer and non integer orders.

The objective of our paper was to replace ordinary derivatives with FF derivatives using kernel of power-law in model (1.1) and compare our findings with the findings of model (1.1) in [6]. We apply results from fixed point theory to establish existence, and convergence of solution of our proposed model. Moreover, we check the stability of our model. Furthermore, we generated Matlab code for our fractal fractional model which described the behavior of nodes for arbitrary values of fractional orders and fractal dimensions to better understand the propagation of malware in-detail to overcome it.

We organized this paper as: in section 2, some basic definitions and theorems are described that are useful for our work. In section 3, fractal fractional mathematical model is formulated. In section 4, this model has been formulated as fixed point problem. Then in sections 5, 6 and 7 existence, uniqueness and stability of the solution is proved by results of fixed point theory. In section 8, a numerical scheme based on two points Lagrangian interpolation is developed as this method provides more accuracy, better convergence and most of all it is simple to understand and coding is easier than other methods. Then coding is done using Matlab R2016a for different fractal and fractional orders and parameters. In section 9, simulation is discussed and in section 10, we concluded our work.

## 2 Preliminaries

### 2.1 Classical calculus

This section includes some definitions and theorems from fixed point theory and fractional calculus which are needed in the sequel. We use the following results defined in [24].

Let Ψ displays a subclass of non-decreasing operators *ψ*: [0, ∞) → [0, ∞) such that

$$\sum_{j=1}^{\infty }\psi^{j}\left(\sigma \right)<\infty \;\forall \;\;\sigma >0,$$

where *ψ*^*j*^ is the j^th^ iterate. It satisfies the following lemma:

**Lemma 2.1**. [24] *Every function ψ*: [0, ∞) → [0, ∞) *in the class* Ψ *satisfies the condition*:

*For any σ* > 0,

$$\underset{j\to \infty }{\text{lim}}\;\psi^{j}\left(\sigma \right)=0\Rightarrow \psi \left(\sigma \right)<\sigma .$$

**Definition 2.2**. [24] *Let* Y *be a normed space and* F:Y→Y *with ψ*: [0, ∞) → [0, ∞) *and* $\phi :Y^{2}\to \left[0,\infty \right)$, *then F is a ϕ* − *ψ* − ***contraction*** *if for* $u_{1},u_{2}\in Y$,

$$\phi \left(u_{1},u_{2}\right)\cdot d\left(F_{u_{1}},F_{u_{2}}\right)\leq \psi \left(d\left(u_{1},u_{2}\right)\right).$$

**Definition 2.3**. [24] *If* F:Y→Y *and* $\phi :Y^{2}\to \left[0,\infty \right)$, *then F is ϕ-admissible if for* $u_{1},u_{2}\in Y$,

$$\phi \left(u_{1},u_{2}\right)\geq 1\Rightarrow \phi \left(F_{u_{1}},F_{u_{2}}\right)\geq 1.$$

With the help of above Definitions 2.2 and 2.3, the authors derived the following results for existence of fixed point.

**Theorem 2.4**. [24] *Let* (Y,d) *be a complete metric space and* F:Y→Y *be a ϕ-ψ-contraction mapping with the conditions*:

*(i) F is ϕ-admissible*;
*(ii) there exists* $y_{0}\in Y$ *such that ϕ*(*y*_0_, *Fy*_0_) ≥ 1;
*(iii) if* {*y*_*n*_} *is a sequence in* Y *such that ϕ*(*y*_*n*_, *y*_*n*+1_) ≥ 1 *for all n and* $y_{n}\to y\in Y$ *as n* → ∞, *implies ϕ*(*y*_*n*_, *y*) ≥ 1 *for all* n∈N.

*Then, F has a fixed point*.

For the existence of solution in the support of (2.4), Leray Schauder criteria is also used which is defined as:

**Theorem 2.5**. [25] *Let* Y *be a Banach space*, E *be a bounded and closed set in* Y *such that* E *is convex and U be an open set in* E *with the property* 0 ∈ *U, then a compact and continuous operator* $G:\bar{U}\to E$, *shows either*

*(a) G has a fixed point in* $\bar{U},$

*or*
*(b) there exists y* ∈ ∂*U and η* ∈ (0, 1) *s.t y* = *ηG*(*y*).

Moreover, for compactness of operator, Arzela-Ascoli’s theorem is used which is defined as:

**Theorem 2.6**. [26] *Let* $V\subset R^{n}$, $W\;\subset \;C(V,R^{m})$. *Then W is compact if and only if W is closed, bounded and equicontinuous*.

### 2.2 Fractional calculus

Now-a-days fractal theory is most widely used to understand the behaviour of variables. To study the behaviour of a given variable with respect to a scaled variable, Chen et al. [27] defined:

**Definition 2.7**. *Fractal derivative (earlier defined as Hausdorff derivative in* [28]) *of f*(*σ*) *with respect to a fractal order* p∈(0,1) *is*

$$\frac{df(\sigma )}{d\sigma^{p}}=\underset{\sigma \to \sigma_{1}}{\text{lim}}\;\frac{f\left(\sigma \right)-f\left(\sigma_{1}\right)}{\sigma^{p}-\sigma_{1}^{p}}.$$

Combining the concepts of fractal differentiation and fractal derivative, a novel concept of differentiation was presented by Atangana [20] as:

**Definition 2.8**. *Let* Υ(*σ*) *be continuous and fractal differentiable of order* p *on* (*a*, *b*). *Then Riemann-liouville fractal-fractional derivative with power law kernel of* Υ *of order* q *is defined as*

DFFPa,σq,pϒ(σ)=1Γ(n-q)ddσp∫aσ(σ-u)n-q-1ϒ(u)du,

*where* (n-1<p,q≤n),n∈N.

He constructed fractal-fractional integral associated to fractal-fractional derivative as:

**Definition 2.9**. [20] *Let* Υ(*σ*) *be continuous on* (*a*, *b*), *then fractal-fractional integral of* Υ *with order* q *and taking n* = 1 *is*

IFFPa,σq,pϒ(σ)=pΓ(q)∫atu(p-1)(σ-u)q-1ϒ(u)du.

## 3 Conversion of classical mathematical model to fractal-fractional mathematical model

Inspired by the concepts of fractal fractional calculus, we convert classical mathematical model (1.1) in terms of fractal-fractional derivatives. So, our required model is

DFFP0,σq,pΔ(σ)=Πθ-β0f(ℵ(σ))Δ(σ)-(μ+ν)Δ(σ)+ζΘ(σ-τ),DFFP0,σq,pℵ(σ)=β0f(ℵ(σ))Δ(σ)-(μ+κ)ℵ(σ),DFFP0,σq,pΘ(σ)=(1-Π)θ+νΔ(σ)+κℵ(σ)-ζΘ(σ-τ)-μΘ(σ), (3.1)

with the initial conditions Δ(0) = Δ_0_ ≥ 0, ℵ(0) = ℵ_0_ ≥ 0, Θ(0) = Θ_0_ ≥ 0, and *N*(*σ*) = Δ(*σ*) + ℵ(*σ*) + Θ(*σ*) for *σ* ∈ *J* = [0, *T*], *T* > 0. Also p,q∈(0,1] and all parameters are to be taken non-negative. Here *FFP* represents the term fractal-fractional derivative with power law kernel defined in [20].

## 4 Formulation of model as fixed point problem

In this section, we convert mathematical model of ODES (1.1) in fixed point problem. We apply results of fixed point theory on model (3.1). Consider $\Xi =Y^{3}$, a Banach space and Y=C(J,R) represents the class of all continuous functions with the norm defined by

$${\left|||Ϝ|\right|}_{\Xi }={\left||\left(\Delta ,ℵ,\Theta \right)|\right|}_{\Xi }=max\{|\Delta (\sigma )|+|ℵ(\sigma )|+|\Theta (\sigma )|:\sigma \in J\}.$$

First, rewrite given model (3.1) as

$$\begin{array}{c} \begin{array}{cc} {ϒ}_{1}\left(\sigma ,\Delta \left(\sigma \right),ℵ\left(\sigma \right),\Theta \left(\sigma \right)\right) & =\Pi \theta -\beta_{0}f\left(ℵ\left(\sigma \right)\right)\Delta \left(\sigma \right)-\left(\mu +\nu \right)\Delta \left(\sigma \right)+\zeta \Theta \left(\sigma -\tau \right),\\ {ϒ}_{2}\left(\sigma ,\Delta \left(\sigma \right),ℵ\left(\sigma \right),\Theta \left(\sigma \right)\right) & =\beta_{0}f\left(ℵ\left(\sigma \right)\right)\Delta \left(\sigma \right)-\left(\mu +\kappa \right)ℵ\left(\sigma \right),\\ {ϒ}_{3}\left(\sigma ,\Delta \left(\sigma \right),ℵ\left(\sigma \right),\Theta \left(t\right)\right) & =(1-\Pi )\theta +\nu \Delta (\sigma )+\kappa I(\sigma )-\zeta \Theta (\sigma -\tau )-\mu \Theta (\sigma ). \end{array} \end{array}$$ (4.1)

Comparing models (3.1) and (4.1), we have

DFFP0,σq,pΔ(σ)=ϒ1(σ,Δ(σ),ℵ(σ),Θ(σ)),DFFP0,σq,pℵ(σ)=ϒ2(σ,Δ(σ),ℵ(σ),Θ(σ)),DFFP0,σq,pΘ(σ)=ϒ3(σ,Δ(σ),ℵ(σ),Θ(σ)). (4.2)

Since

DFFP0,σq,pg(t)=1Γ(1-q)ddσp∫0σ(σ-u)-qg(u)du

DFFP0,σq,pg(σ)=1Γ(1-q)1pσp-1ddσ∫0σ(σ-u)-qg(u)du

DFFP0,σq,pg(σ)=(1pσp-1)1Γ(1-q)ddσ∫0σ(σ-u)-qg(u)du

DFFP0,σq,pg(σ)=(1pσp-1)RLD0,σqg(σ),

where from ([8]) for *n* = 1, we have

$$\frac{1}{\Gamma (1-q)}\frac{d}{d\sigma }\int_{0}^{\sigma }{\left(\sigma -u\right)}^{-q}\;g\left(u\right)du{=}^{RL}D_{0,\sigma }^{q}g\left(\sigma \right).$$

So, model (4.2) can be written as

$$\begin{array}{c} \begin{array}{cc} {\left(\frac{1}{p\sigma^{p-1}}\right)}^{RL}D_{0,\sigma }^{q}\Delta \left(\sigma \right) & ={ϒ}_{1}\left(\sigma ,\Delta \left(\sigma \right),ℵ\left(\sigma \right),\Theta \left(\sigma \right)\right),\\ {\left(\frac{1}{p\sigma^{p-1}}\right)}^{RL}D_{0,\sigma }^{q}ℵ\left(\sigma \right) & ={ϒ}_{2}\left(\sigma ,\Delta \left(\sigma \right),ℵ\left(\sigma \right),\Theta \left(\sigma \right)\right),\\ {\left(\frac{1}{p\sigma^{p-1}}\right)}^{RL}D_{0,\sigma }^{q}\Theta \left(\sigma \right) & ={ϒ}_{3}\left(\sigma ,\Delta \left(\sigma \right),ℵ\left(\sigma \right),\Theta \left(\sigma \right)\right). \end{array} \end{array}$$ (4.3)

Hence, we get

DRL0,σqΔ(σ)=pσp-1ϒ1(σ,Δ(σ),ℵ(σ),Θ(σ)),DRL0,σqℵ(σ)=pσp-1ϒ2(σ,Δ(σ),ℵ(σ),Θ(σ)),DRL0,σqΘ(σ)=pσp-1ϒ3(σ,Δ(σ),ℵ(σ),Θ(σ)). (4.4)

In general, we can write model (4.4) as

DRL0,σqϜ(σ)=pσp-1ϒ(σ,Ϝ(σ)),Ϝ(0)=Ϝ0, (4.5)

where

$$\begin{array}{c} \begin{array}{cc} & (p,q)\in (0,1],\\ & \sigma \in J,\\ Ϝ(\sigma ) & ={\left(\Delta \left(\sigma \right),ℵ\left(\sigma \right),\Theta \left(\sigma \right)\right)}^{⊤},\\ {Ϝ}_{0} & ={\left(\Delta_{0},{ℵ}_{0},\Theta_{0}\right)}^{⊤}. \end{array} \end{array}$$

Applying fractal-fractional integral on model (4.5), using the result in ([20]), we get

$$\begin{array}{c} Ϝ\left(\sigma \right)-Ϝ\left(0\right)=\frac{p}{\Gamma (q)}\int_{0}^{\sigma }u^{\left(p-1\right)}\;{\left(\sigma -u\right)}^{q-1}\;ϒ\left(u,Ϝ\left(u\right)\right)du. \end{array}$$ (4.6)

Hence, we can write

$$\begin{array}{c} \begin{array}{cc} \Delta (\sigma ) & =\Delta \left(0\right)+\frac{p}{\Gamma (q)}\int_{0}^{\sigma }u^{\left(p-1\right)}\;{\left(\sigma -u\right)}^{q-1}\;{ϒ}_{1}\left(u,\Delta \left(u\right),I\left(u\right),\Theta \left(u\right)\right)du,\\ ℵ(\sigma ) & =ℵ\left(0\right)+\frac{p}{\Gamma (q)}\int_{0}^{\sigma }u^{\left(p-1\right)}\;{\left(\sigma -u\right)}^{q-1}\;{ϒ}_{2}\left(u,\Delta \left(u\right),ℵ\left(u\right),\Theta \left(u\right)\right)du,\\ \Theta (\sigma ) & =\Theta \left(0\right)+\frac{p}{\Gamma (q)}\int_{0}^{\sigma }u^{\left(p-1\right)}\;{\left(\sigma -u\right)}^{q-1}\;{ϒ}_{3}\left(u,\Delta \left(u\right),ℵ\left(u\right),\Theta \left(u\right)\right)du. \end{array} \end{array}$$ (4.7)

So, now we can transform (3.1) into a fixed point problem.

Define an operator *F*: Ξ → Ξ by

$$\begin{array}{c} F\left(Ϝ\left(\sigma \right)\right)=Ϝ\left(0\right)+\frac{p}{\Gamma (q)}\int_{0}^{\sigma }u^{\left(p-1\right)}\;{\left(\sigma -u\right)}^{q-1}\;ϒ\left(u,Ϝ\left(u\right)\right)du. \end{array}$$ (4.8)

## 5 Existence of solution

For existence, we prove a theorem on the basis of Theorem 2.4 as in ([30]).

**Theorem 5.1**. *Suppose that* ∃ $V:R^{3}\times R^{3}\to R$, *ψ* ∈ Ψ *and* Υ ∈ *C*(*J* × Ξ, Ξ) *satisfying the following conditions*:

(*β*_1_): $\forall {Ϝ}_{1},{Ϝ}_{2}\in \Xi$ *and σ* ∈ *J*,

$$\begin{array}{c} |ϒ\left(\sigma ,{Ϝ}_{1}\left(\sigma \right)\right)-ϒ\left(\sigma ,{Ϝ}_{2}\left(\sigma \right)\right)\left|\leq \ell \;\psi (\right|{Ϝ}_{1}\left(\sigma \right)-{Ϝ}_{2}\left(\sigma \right)\left|\right), \end{array}$$

*with* $V({Ϝ}_{1}\left(\sigma \right),{Ϝ}_{2}\left(\sigma \right))\geq 0$ *and* $\ell =\frac{\Gamma (p+q)}{p\;\;T^{\left(p+q-1\right)}\;\Gamma \left(p\right)}$;

(*β*_2_): $\exists \;\;{Ϝ}_{0}\in \Xi$ *such that* ∀ *σ* ∈ *J*,

$$\begin{array}{cc} & V({Ϝ}_{0}\left(\sigma \right),F\left({Ϝ}_{0}\left(\sigma \right)\right))\geq 0,\\ & \text{and}\\ & V({Ϝ}_{1}\left(\sigma \right),{Ϝ}_{2}\left(\sigma \right))\geq 0\\ & gives \end{array}$$

$$\begin{array}{c} V(F\left({Ϝ}_{1}\left(\sigma \right)\right),F\left({Ϝ}_{2}\left(\sigma \right)\right))\geq 0\;\;; \end{array}$$

(*β*_3_): $\forall {\left\{{Ϝ}_{n}\right\}}_{n\geq 1}\subseteq \Xi$ *with* ${Ϝ}_{n}\to Ϝ$,

$$\begin{array}{c} V\left({Ϝ}_{n}\left(\sigma \right),{Ϝ}_{n+1}\left(\sigma \right)\right)\geq 0\Rightarrow V\left({Ϝ}_{n}\left(\sigma \right),Ϝ\left(\sigma \right)\right)\geq 0, \end{array}$$ (5.1)

*for every n and σ*.

*Hence, we say that F has a fixed point. So a solution of malware propagation model exists*.

*Proof*. Take ${Ϝ}_{1},{Ϝ}_{2}\in \Xi$ so that

$$\begin{array}{c} V({Ϝ}_{1}\left(\sigma \right),{Ϝ}_{2}\left(\sigma \right))\geq 0, \end{array}$$

for every *σ* ∈ *J*.

Now, we take

$$\begin{array}{ccc} |F\left({Ϝ}_{1}\left(\sigma \right)\right)-F\left({Ϝ}_{2}\left(\sigma \right)\right)| & = & |\frac{p}{\Gamma (q)}\int_{0}^{\sigma }u^{\left(p-1\right)}\;{\left(\sigma -u\right)}^{q-1}\;\left(ϒ\left(u,{Ϝ}_{1}\left(u\right)\right)-ϒ\left(u,{Ϝ}_{2}\left(u\right)\right)\right)\;du|\\ & \leq & \frac{p}{\Gamma (q)}\int_{0}^{\sigma }u^{\left(p-1\right)}\;{\left(\sigma -u\right)}^{q-1}\;\left|ϒ\left(u,{Ϝ}_{1}\left(u\right)\right)-ϒ\left(u,{Ϝ}_{2}\left(u\right)\right)\right|\;du. \end{array}$$

By using (*β*_1_), we deduce

$$\begin{array}{ccc} |F\left({Ϝ}_{1}\left(\sigma \right)\right)-F\left({Ϝ}_{2}\left(\sigma \right)\right)| & \leq & \frac{p}{\Gamma (q)}\int_{0}^{\sigma }u^{\left(p-1\right)}\;{\left(\sigma -u\right)}^{q-1}\;\ell \;\psi (|{Ϝ}_{1}\left(u\right)-{Ϝ}_{2}\left(u\right)\left|\right)\;du. \end{array}$$

Now, by using the definition of norm

$$\begin{array}{ccc} |F\left({Ϝ}_{1}\left(\sigma \right)\right)-F\left({Ϝ}_{2}\left(\sigma \right)\right)| & \leq & \frac{p}{\Gamma (q)}\int_{0}^{\sigma }u^{\left(p-1\right)}\;{\left(\sigma -u\right)}^{q-1}\;\ell \;\psi (||{Ϝ}_{1}-{Ϝ}_{2}{\left|\right|}_{\Xi })\;du. \end{array}$$

After doing some computations and using the definition of beta function and using definition of *ℓ*, we get

$$\begin{array}{c} ||F\left({Ϝ}_{1}\left(\sigma \right)\right)-F\left({Ϝ}_{2}\left(\sigma \right)\right){\left|\right|}_{\Xi }\leq \;\psi (||{Ϝ}_{1}-{Ϝ}_{2}{\left|\right|}_{\Xi }). \end{array}$$ (5.2)

Moreover, if we define a function *ϕ*: Ξ^2^ → [0, ∞) such that $\phi ({Ϝ}_{1},{Ϝ}_{2})=1$ for $V({Ϝ}_{1}\left(\sigma \right),{Ϝ}_{2}\left(\sigma \right))\geq 0$, and zero otherwise, then for each ${Ϝ}_{1},{Ϝ}_{2}\in \Xi$ equation (5.2) can be written as

$$\begin{array}{c} \phi \left({Ϝ}_{1},{Ϝ}_{2}\right)\;d\left(F\left({Ϝ}_{1}\right),F\left({Ϝ}_{2}\right)\right)\leq \;\psi \left(d\left({Ϝ}_{1},{Ϝ}_{2}\right)\right). \end{array}$$

This shows that *F* is a *ϕ*-*ψ*-*contraction*.

Now, suppose that ${Ϝ}_{1},{Ϝ}_{2}\in \Xi$ with the property that $\phi ({Ϝ}_{1},{Ϝ}_{2})\geq 1$. By the definition of *ϕ*, we deduce

$$\begin{array}{c} V({Ϝ}_{1}\left(\sigma \right),{Ϝ}_{2}\left(\sigma \right))\geq 0, \end{array}$$

and by (*β*_2_), we get $V({Ϝ}_{0}\left(\sigma \right),F\left({Ϝ}_{0}\left(\sigma \right)\right))\geq 0$ and $V({Ϝ}_{1}\left(\sigma \right),{Ϝ}_{2}\left(\sigma \right))\geq 0,\Rightarrow V(F\left({Ϝ}_{1}\left(\sigma \right)\right),F\left({Ϝ}_{2}\left(\sigma \right)\right))\geq 0$.

So, by applying definition of *ϕ*, we have

$$\begin{array}{c} \phi (F\left({Ϝ}_{1}\right),F\left({Ϝ}_{2}\right))\geq 1. \end{array}$$

Hence, *F* is *ϕ*-*admissible*. (*)

Moreover, by (*β*_2_), it can be seen that for some ${Ϝ}_{0}$ in Ξ, ∀*σ* ∈ *J*, we have $V({Ϝ}_{0}\left(\sigma \right),F\left({Ϝ}_{0}\left(\sigma \right)\right))\geq 0\Rightarrow \phi ({Ϝ}_{0},F\left({Ϝ}_{0}\right))\geq 1$. (**)

Now, consider ${\left\{{Ϝ}_{n}\right\}}_{n\geq 1}\subseteq \Xi$ with ${Ϝ}_{n}\to Ϝ$ and for all *n* and $\phi ({Ϝ}_{n},{Ϝ}_{n+1})\geq 1.$

By definition of *ϕ* this implies $V({Ϝ}_{n}\left(\sigma \right),{Ϝ}_{n+1}\left(\sigma \right))\geq 0$.

Thus, by (*β*_3_) this implies $V({Ϝ}_{n}\left(\sigma \right),Ϝ\left(\sigma \right))\geq 0.$

Hence, $\phi ({Ϝ}_{n},Ϝ)\geq 1$ for all *n*. (***)

So (*), (**), (***) show the conditions of Theorem 2.4 are satisfied, so we can say that there exists some ${Ϝ}^{*}\in \Xi$ such that $F\left({Ϝ}^{*}\right)={Ϝ}^{*}$.

Hence, ${Ϝ}^{*}={\left(\Delta^{*},{ℵ}^{*},\Theta^{*}\right)}^{⊤}$ is a solution of our model.

Theorem 2.5 also establishes that solution of model exists and on basis of this model we also define the following theorem as:

**Theorem 5.2**. *Let* Ξ *be a Banach space*, $N_{\epsilon }$ *be a bounded and closed set in* Ξ *and A be an open in* $N_{\epsilon }$ *with* 0 ∈ *A, then there exists a compact and continuous operator F with the conditions* (*β*_4_) *and* (*β*_5_) *from* $\bar{A}\to N_{\epsilon }$ *which satisfies one of the two conditions*,

*(a) G has a fixed point in* $\bar{A}$ ,

*or*

*(b) there exists* Ϝ∈∂A *and ω* ∈ (0, 1) *s.t* Ϝ=ωF(Ϝ);

*where*

(*β*_4_): *Suppose* Υ ∈ *C*(*J* × Ξ, Ξ) *and there exists ϕ* ∈ *L*^1^(*J*, [0, ∞)) *and B* ∈ *C*([0, ∞), [0, ∞)) *where B is an increasing function satisfying the condition* |F(σ,Ϝ(σ))|≤ϕ(σ)B(|Ϝ(σ)|) *for all σ* ∈ *J and* Ϝ∈Ξ;

(*β*_5_): If ϕ* = *sup*_*σ* ∈ *J*_|*ϕ*(*σ*)| *then* ∃ *a no r s.t* $\frac{r}{{Ϝ}_{0}+\lambda \;\phi^{*}\;B\left(r\right)}>1$ *where* $\lambda =\frac{p\;T^{p+q-1}\;\Gamma \left(p\right)}{\Gamma (p+q)}$.

*Proof*. Consider *F*: Ξ → Ξ *as*

$$F\left(Ϝ\left(\sigma \right)\right)=Ϝ\left(0\right)+\frac{p}{\Gamma (q)}\int_{0}^{\sigma }u^{\left(p-1\right)}\;{\left(\sigma -u\right)}^{q-1}\;ϒ\left(u,\left(Ϝu\right)\right)du,$$

and $N_{\epsilon }={\left\{Ϝ\in \Xi :||Ϝ|\right|}_{\Xi }\leq \epsilon \}$ for some positive *ϵ*.

We show that *F* is compact on $N_{\epsilon }$. For this we prove that *F* is uniformly bounded and equicontinuous.

Since Υ is continuous, this implies *F* is continuous.

Now for Ϝ in $N_{\epsilon }$, we obtain

$$\left|F\left(Ϝ\left(\sigma \right)\right)\right|\leq \left|Ϝ\left(0\right)\right|+\frac{p}{\Gamma (q)}\int_{0}^{\sigma }u^{\left(p-1\right)}\;{\left(\sigma -u\right)}^{q-1}\;\left|ϒ\left(u,Ϝ\left(u\right)\right)\right|du$$

and from (*β*_4_), we have

$$\begin{array}{ccc} \left|F(Ϝ(\sigma ))\right| & \leq & {Ϝ}_{0}+\frac{p}{\Gamma (q)}\int_{0}^{\sigma }u^{\left(p-1\right)}\;{\left(\sigma -u\right)}^{q-1}\;\phi \left(u\right)\;B\left(|Ϝ\left(u\right)|\right)du\\ & \leq & {Ϝ}_{0}+\frac{p}{\Gamma (q)}\int_{0}^{\sigma }u^{\left(p-1\right)}\;{\left(\sigma -u\right)}^{q-1}\;\phi^{*}{\;B(||Ϝ||}_{\Xi })du\\ & \leq & {Ϝ}_{0}+\frac{p}{\Gamma (q)}\;\phi^{*}{\;B(||Ϝ||}_{\Xi })\;\int_{0}^{\sigma }u^{\left(p-1\right)}\;{\left(\sigma -u\right)}^{q-1}\;du, \end{array}$$

after simplification of the integral, we get the beta function. So applying value of beta function and λ, we get

$$\left|F\left(Ϝ\left(\sigma \right)\right)\right|\leq {Ϝ}_{0}+\lambda \;\phi^{*}\;B\left(\epsilon \right).$$

Hence, by applying norm, we have

$$\begin{array}{c} \left||F\left(Ϝ\left(\sigma \right)\right)|\right|\leq {Ϝ}_{0}+\lambda \;\phi^{*}\;B\left(\epsilon \right)<\infty . \end{array}$$ (5.3)

This implies *F* is uniformly bounded.

Now, for *σ*, σ* ∈ *J* such that *σ* < σ* and $Ϝ\in N_{\epsilon }$ arbitrarily.

If we suppose ${ϒ}^{*}\;=\;sup\;\left|ϒ\left(\sigma ,Ϝ\left(\sigma \right)\right)\right|$, then

$$\begin{array}{ccc} |F\left(Ϝ\left(\sigma^{*}\right)\right)-F\left(Ϝ\left(\sigma \right)\right)| & = & |\frac{p}{\Gamma (q)}\int_{0}^{\sigma^{*}}u^{\left(p-1\right)}\;{\left(\sigma^{*}-u\right)}^{q-1}\;ϒ\left(u,Ϝ\left(u\right)\right)\;du\\ & - & \frac{p}{\Gamma (q)}\int_{0}^{\sigma }u^{\left(p-1\right)}\;{\left(\sigma -u\right)}^{q-1}\;ϒ\left(u,Ϝ\left(u\right)\right)\;du|\\ & \leq & \frac{p}{\Gamma (q)}|\int_{0}^{\sigma^{*}}u^{\left(p-1\right)}\;{\left(\sigma^{*}-u\right)}^{q-1}\;du\\ & - & \int_{0}^{\sigma }u^{\left(p-1\right)}\;{\left(\sigma -u\right)}^{q-1}\;du\;|\;\cdot |ϒ(u,Ϝ\left(u\right)|\\ & \leq & \frac{p}{\Gamma (q)}\left|{\left(\sigma^{*}\right)}^{\left(p+q-1\right)}\;\beta \left(p,q\right)-\sigma^{\left(p+q-1\right)}\;\beta \left(p,q\right)\right|\;{ϒ}^{*}\\ & \leq & \frac{p}{\Gamma (p+q)}\;{ϒ}^{*}\;\left[{\left(\sigma^{*}\right)}^{\left(p+q-1\right)}-\sigma^{\left(p+q-1\right)}\right], \end{array}$$

that is independent from Ϝ. When σ* → *σ*, its value becomes zero. Hence $||F\left(Ϝ\left(\sigma^{*}\right)\right)-F\left(Ϝ\left(\sigma \right)\right){\left|\right|}_{\Xi }\to 0$. Thus proved that *F* is equicontinuous. So *F* is compact. As *F* satisfies the conditions of Theorem 5.2, we say that *F* will satisfy either one or the other conditions mentioned in Theorem 5.2. For this using (*β*_5_), we construct $A=\{Ϝ\in \Xi :||Ϝ|{|}_{\Xi }<r\},$ where *r* > 0 is defined above. Hence, we can write

$$\begin{array}{c} \left||F\left(Ϝ\left(\sigma \right)\right)|\right|\leq {Ϝ}_{0}+\lambda \;\phi^{*}\;B\left(r\right). \end{array}$$ (5.4)

Assume, there exists Ϝ∈∂A and *ω* ∈ (0, 1) where Ϝ=ωF(Ϝ).

For Ϝ, *ω* and using (*β*_5_), we get

$$\begin{array}{ccc} r & = & {\left||Ϝ|\right|}_{\Xi }\\ & = & {\omega ||F\left(Ϝ\right)||}_{\Xi }\\ & < & {\left||F\left(Ϝ\right)|\right|}_{\Xi }\\ & < & {Ϝ}_{0}+\lambda \;\phi^{*}{\;B(||Ϝ||}_{\Xi })\\ & < & {Ϝ}_{0}+\lambda \;\phi^{*}\;B\left(r\right). \end{array}$$

This gives us *r* < *r*, which is impossible. Thus, condition (*b*) is not satisfied. Hence, by condition (*a*), *F* possesses a fixed point in $\bar{A}$.

## 6 Uniqueness

Now we will prove uniqueness with the help of theorems using lipschitz condition [34] along with some other conditions.

**Theorem 6.1**. *Let* $\Delta ,ℵ,\Theta ,\Delta_{1},{ℵ}_{1},\Theta_{1}\in Y=C\left(J,R\right)$ *and we assume that* **(*Condition*)**: ||Δ|| ≤ *μ*_1_, ||ℵ|| ≤ *μ*_2_, *α* ∈ (0, ∞), $||f\left(ℵ\left(\sigma \right)\right)||\;=\;||\frac{ℵ(\sigma )}{1+\alpha \;ℵ(t)}\left|\right|\;\;\leq \frac{\left||ℵ(\sigma )|\right|}{\left||1+\alpha \;ℵ(\sigma )|\right|}\;\leq \;\mu_{3}$ (*where* $\;\mu_{3}=\frac{1}{\alpha }),$ ||Θ|| ≤ *μ*_4_ *for some* *μ*_*i*_ > 0, *i* = 1, 2, 3, 4.

*Moreover* $\left|\right|\frac{1}{1+\alpha \;ℵ(\sigma )}\left|\right|\;\leq b_{1}$ , $\left|\right|\frac{1}{1+\alpha \;{ℵ}_{1}\left(\sigma \right)}\left|\right|\;\leq \;b_{2},$ *where* $b_{1}=\;\frac{1}{\alpha ||ℵ(t)||}$, $b_{2}=\;\frac{1}{\alpha ||{ℵ}_{1}\left(\sigma \right)\left|\right|}$ *and b*_1_ > 0, *b*_2_ > 0 *and b* = *b*_1_ ⋅ *b*_2_, *then* Υ_1_, Υ_2_, Υ_3_ *defined in Model 4.1 are lipschitz functions with the following values* *w*_1_ = (*β*_0_ *μ*_3_ + *μ* + *ν*), *w*_2_ = (*β*_0_ *μ*_1_ *b* + *μ* + *γ*), *w*_3_ = (*ζ* + *μ*), *where* 0 < *w*_*i*_ < 1, *i* = 1, 2, 3.

*Proof*. Considering Υ_1_ for each $\Delta ,\Delta_{1}\in Y$, we take

$$\begin{array}{ccc} & \left|\right| & {ϒ}_{1}\left(\sigma ,\Delta \left(\sigma \right),ℵ\left(\sigma \right),\Theta \left(\sigma \right)\right)-{ϒ}_{1}\left(\sigma ,\Delta_{1}\left(\sigma \right),ℵ\left(\sigma \right),\Theta \left(\sigma \right)\right)\left|\right|\;\\ & = & \left||(\Pi \theta -\beta_{0}f\left(ℵ\left(\sigma \right)\right)\Delta \left(\sigma \right)-\left(\mu +\nu \right)\Delta \left(\sigma \right)+\zeta \;\Theta \left(\sigma -\tau \right)\right)\\ & - & \left(\Pi \theta -\beta_{0}f\left(ℵ\left(\sigma \right)\right)\Delta_{1}\left(\sigma \right)-\left(\mu +\nu \right)\Delta_{1}\left(\sigma \right)+\zeta \Theta \left(\sigma -\tau \right))|\right|\\ & = & \left|\right|-\beta_{0}f\left(ℵ\left(\sigma \right)\right)\left(\Delta \left(\sigma \right)-\Delta_{1}\left(\sigma \right)\right)-\left(\mu +\nu \right)\left(\Delta \left(\sigma \right)-\Delta_{1}\left(\sigma \right)\right)\left|\right|\\ & = & \left|\right|\left(-\beta_{0}f\left(ℵ\left(\sigma \right)\right)-\left(\mu +\nu \right)\right)\left(\Delta \left(\sigma \right)-\Delta_{1}\left(\sigma \right)\right)\left|\right|\\ & = & \left|\right|\left(-\left(\beta_{0}f\left(ℵ\left(\sigma \right)\right)+\left(\mu +\nu \right)\right)\right)\left(\Delta \left(\sigma \right)-\Delta_{1}\left(\sigma \right)\right)\left|\right|\\ & = & \left|\right|\left(\beta_{0}f\left(ℵ\left(\sigma \right)\right)+\left(\mu +\nu \right)\right)\left(\Delta \left(\sigma \right)-\Delta_{1}\left(\sigma \right)\right)\left|\right|\\ & \leq & \left(|\right|\left(\beta_{0}f\left(ℵ\left(\sigma \right)\right)+\left(\mu +\nu \right)\right)||)\;||\Delta \left(\sigma \right)-\Delta_{1}\left(\sigma \right)\left|\right|\\ & \leq & (||(\beta_{0}f\left(ℵ\left(\sigma \right)\right)||+||\left(\mu +\nu \right)||)\;||\Delta \left(\sigma \right)-\Delta_{1}\left(\sigma \right)\left|\right|\\ & \leq & \left(\beta_{0}\;\mu_{3}+\mu +\nu \right)\;||\Delta \left(\sigma \right)-\Delta_{1}\left(\sigma \right)\left|\right|\\ & \leq & w_{1}\;||\Delta \left(\sigma \right)-\Delta_{1}\left(\sigma \right)\left|\right|. \end{array}$$

Hence, Υ_1_ is Lipschitz with respect to Δ with *w*_1_ > 0.

Consider Υ_2_ for each $ℵ,{ℵ}_{1}\in Y$, we take

$$\begin{array}{ccc} & \left|\right| & {ϒ}_{2}\left(\sigma ,\Delta \left(\sigma \right),ℵ\left(\sigma \right),\Theta \left(\sigma \right)\right)-{ϒ}_{2}\left(\sigma ,\Delta \left(\sigma \right),{ℵ}_{1}\left(\sigma \right),\Theta \left(\sigma \right)\right)\;\left|\right|\\ & = & \left|\right|\left(\beta_{0}f\left(ℵ\left(\sigma \right)\right)\Delta \left(\sigma \right)-\left(\mu +\gamma \right)ℵ\left(\sigma \right)\right)-\left(\beta_{0}f\left({ℵ}_{1}\left(\sigma \right)\right)\Delta \left(\sigma \right)-\left(\mu +\gamma \right){ℵ}_{1}\left(\sigma \right)\right)\left|\right|\\ & = & \left||(\beta_{0}\Delta \left(\sigma \right)\left(f\left(ℵ\left(\sigma \right)\right)-f\left({ℵ}_{1}\left(\sigma \right)\right)\right)+\left(\mu +\gamma \right)\left(-ℵ\left(\sigma \right)+{ℵ}_{1}\left(\sigma \right)\right)|\right|\\ & \leq & \left||(\beta_{0}\Delta \left(\sigma \right)\left(f\left(ℵ\left(\sigma \right)\right)-f\left({ℵ}_{1}\left(\sigma \right)\right)\right)||+||\left(\mu +\gamma \right)\left(-ℵ\left(\sigma \right)+{ℵ}_{1}\left(\sigma \right)\right)|\right|\\ & \leq & |\beta_{0}\left|\;||\Delta \left(\sigma \right)||\;|\right|\left(f\left(ℵ\left(\sigma \right)\right)-f\left({ℵ}_{1}\left(\sigma \right)\right)\right)\left|\right|+\left|\left(\mu +\gamma \right)|\;|\right|\left(ℵ\left(\sigma \right)-{ℵ}_{1}\left(\sigma \right)\right)\left|\right|\\ & \leq & \beta_{0}\;\mu_{1}\;||\frac{ℵ(\sigma )}{1+\alpha \;ℵ(\sigma )}-\frac{{ℵ}_{1}\left(\sigma \right)}{1+\alpha \;{ℵ}_{1}\left(\sigma \right)}\left|\right|+\left(\mu +\gamma \right)||ℵ\left(\sigma \right)-{ℵ}_{1}\left(\sigma \right))||\\ & \leq & \beta_{0}\;\mu_{1}\;||ℵ\left(\sigma \right)-{ℵ}_{1}\left(\sigma \right)||\;\frac{1}{||\left(1+\alpha \;ℵ\left(\sigma \right)\right)\;\left(1+\alpha \;{ℵ}_{1}\left(\sigma \right)\right)\left|\right|}+\left(\mu +\gamma \right)||ℵ\left(\sigma \right)-{ℵ}_{1}\left(\sigma \right)\left|\right|\\ & \leq & \beta_{0}\;\mu_{1}\;b\;||ℵ\left(\sigma \right)-{ℵ}_{1}\left(\sigma \right)||+\left(\mu +\gamma \right)||ℵ\left(\sigma \right)-{ℵ}_{1}\left(\sigma \right)\left|\right|\\ & \leq & \left(\beta_{0}\;\mu_{1}\;b+\mu +\gamma \right)||ℵ\left(\sigma \right)-{ℵ}_{1}\left(\sigma \right)\left|\right|\\ & \leq & w_{2}||ℵ\left(\sigma \right)-{ℵ}_{1}\left(\sigma \right)\left|\right|. \end{array}$$

Hence, Υ_2_ is Lipschitz w.r.t ℵ with *w*_2_ > 0.

Considering Υ_3_ for each $\Theta ,\Theta_{1}\in Y$, we take

$$\begin{array}{ccc} & \left|\right| & {ϒ}_{3}\left(\sigma ,\Delta \left(\sigma \right),ℵ\left(\sigma \right),\Theta \left(\sigma \right)\right)-{ϒ}_{3}\left(\sigma ,\Delta \left(\sigma \right),{ℵ}_{(}\sigma \right),\Theta_{1}\left(\sigma \right))||\\ & = & \left||((1-\Pi )\theta +\nu \Delta (\sigma )+\kappa ℵ(\sigma )-\zeta \Theta (\sigma -\tau )-\mu \Theta (\sigma )\right)\\ & - & \left(\left(1-\Pi \right)\theta +\nu \Delta \left(\sigma \right)+\kappa ℵ\left(\sigma \right)-\zeta \Theta_{1}\left(\sigma -\tau \right)-\mu \Theta_{1}\left(\sigma \right))|\right|\\ & = & ||\zeta \left(\Theta_{1}\left(\sigma -\tau \right)-\Theta \left(\sigma -\tau \right)\right)+\mu \left(\Theta_{1}\left(\sigma \right)-\Theta \left(\sigma \right)\right)\left|\right|\\ & \leq & ||\zeta \left(\Theta_{1}\left(\sigma -\tau \right)-\Theta \left(\sigma -\tau \right)\right)\left|\right|+||\mu \left(\Theta_{1}\left(\sigma \right)-\Theta \left(\sigma \right)\right)\left|\right|\\ & \leq & |\zeta |\;||\Theta \left(\sigma -\tau \right)-\Theta_{1}\left(\sigma -\tau \right)||+\left|\mu ||\right|\left(\Theta \left(\sigma \right)-\Theta_{1}\left(\sigma \right)\right)\left|\right|\\ & & \text{For}\;\sigma \in J\;\text{and}\;\text{for}\;\tau \geq 0\text{,}\;\text{if}\;(\sigma -\tau )\in J\text{,taking}\;\sigma^{*}=max\left(\sigma ,\sigma -\tau \right)\text{,}\;\text{we}\;\text{have}\\ & \leq & \zeta ||\Theta \left(\sigma^{*}\right)-\Theta_{1}\left(\sigma^{*}\right)\left|\right|+\mu ||\Theta \left(\sigma^{*}\right)-\Theta_{1}\left(\sigma^{*}\right)\left|\right|\\ & \leq & \left(\zeta +\mu \right)||\Theta \left(\sigma^{*}\right)-\Theta_{1}\left(\sigma^{*}\right)\left|\right|\\ & \leq & w_{3}||\Theta \left(\sigma^{*}\right)-\Theta_{1}\left(\sigma^{*}\right)\left|\right| \end{array}$$

Hence, Υ_3_ is Lipschitz with respect to Θ with *w*_3_ > 0.

Moreover, we see the uniqueness of solution in Theorem 6.2 under the condition defined in Theorem 6.1.

**Theorem 6.2**. *If* ||Δ|| ≤ *μ*_1_, ||ℵ|| ≤ *μ*_2_, ||Θ|| ≤ *μ*_4_ *for some μ*_*i*_ > 0, *i* = 1, 2, 3, 4 *and* *w*_1_ = (*β*_0_ *μ*_3_ + *μ* + *ν*), *w*_2_ = (*β*_0_ *μ*_1_ *b* + *μ* + *γ*), *w*_3_ = (*ζ* + *μ*), *where* 0 < *w*_*i*_ < 1, *i* = 1, 2, 3; *then our model has a unique solution if λw*_*i*_ < 1 *for i* = 1, 2, 3.

*Proof*. Suppose the model has two solutions (Δ(*σ*), ℵ(*σ*), Θ(*σ*)) and (Δ*(*σ*), ℵ*(*σ*), Θ*(*σ*)) with initial conditions defined above. So, we can write

$$\begin{array}{ccc} \Delta (\sigma ) & = & \Delta \left(0\right)+\frac{p}{\Gamma (q)}\int_{0}^{\sigma }u^{\left(p-1\right)}\;{\left(\sigma -u\right)}^{q-1}\;{ϒ}_{1}\left(u,\Delta \left(u\right),ℵ\left(u\right),\Theta \left(u\right)\right)du,\\ \Delta^{*}\left(\sigma \right) & = & \Delta \left(0\right)+\frac{p}{\Gamma (q)}\int_{0}^{\sigma }u^{\left(p-1\right)}\;{\left(\sigma -u\right)}^{q-1}\;{ϒ}_{1}\left(u,\Delta^{*}\left(u\right),{ℵ}^{*}\left(u\right),\Theta^{*}\left(u\right)\right)du. \end{array}$$

Take

$$\begin{array}{ccc} ||\Delta \left(\sigma \right)-\Delta^{*}\left(\sigma \right)\left|\right| & = & \left|\right|\frac{p}{\Gamma (q)}\int_{0}^{\sigma }u^{\left(p-1\right)}{\left(\sigma -u\right)}^{q-1}({ϒ}_{1}\left(u,\Delta \left(u\right),ℵ\left(u\right),\Theta \left(u\right)\right)\\ & - & {ϒ}_{1}\left(u,\Delta^{*}\left(u\right),{ℵ}^{*}\left(u\right),\Theta^{*}\left(u\right)\right))du||\\ & \leq & \frac{p}{\Gamma (q)}\int_{0}^{\sigma }u^{\left(p-1\right)}\;{\left(\sigma -u\right)}^{q-1}\;\left|\right|{ϒ}_{1}\left(u,\Delta \left(u\right),ℵ\left(u\right),\Theta \left(u\right)\right)\\ & - & {ϒ}_{1}\left(u,\Delta^{*}\left(u\right),{ℵ}^{*}\left(u\right),\Theta^{*}\left(u\right)\right)\left|\right|du, \end{array}$$

since Υ_1_ is considered with respect to Δ and Δ* so by Theorem 6.1 and definition of Beta function, we get

$$\begin{array}{ccc} ||\Delta \left(\sigma \right)-\Delta^{*}\left(\sigma \right)\left|\right| & \leq & \frac{p}{\Gamma (q)}\int_{0}^{\sigma }u^{\left(p-1\right)}\;{\left(\sigma -u\right)}^{q-1}\;||{ϒ}_{1}\left(\Delta \right)-{ϒ}_{1}\left(\Delta^{*}\right)\left|\right|du\\ & \leq & \frac{p}{\Gamma (q)}\;T^{\left(p+q-1\right)}\;\beta \left(p,q\right)\;||{ϒ}_{1}\left(\Delta \right)-{ϒ}_{1}\left(\Delta^{*}\right)\left|\right|\\ & \leq & \frac{p\;T^{\left(p+q-1\right)}\;\Gamma \left(p\right)}{\Gamma (p+q)}\;w_{1}||\Delta \left(\sigma \right)-\Delta^{*}\left(\sigma \right)\left|\right|. \end{array}$$

Hence,

$$||\Delta \left(\sigma \right)-\Delta^{*}\left(\sigma \right)||\leq \lambda \;w_{1}\;||\Delta \left(\sigma \right)-\Delta^{*}\left(\sigma \right)\left|\right|.$$

This implies that (1 − λ*w*_1_)||Δ(*σ*) − Δ*(*σ*)|| ≤ 0.

As λ*w*_1_ < 1, so this is possible when ||Δ(*σ*) − Δ*(*σ*)|| = 0. Thus, Δ(*σ*) = Δ*(*σ*).

Similarly, we have

$$\begin{array}{ccc} ℵ(\sigma ) & = & ℵ\left(0\right)+\frac{p}{\Gamma (q)}\int_{0}^{\sigma }u^{\left(p-1\right)}\;{\left(\sigma -u\right)}^{p-1}\;{ϒ}_{2}\left(u,\Delta \left(u\right),ℵ\left(u\right),\Theta \left(u\right)\right)du,\\ {ℵ}^{*}\left(\sigma \right) & = & ℵ\left(0\right)+\frac{p}{\Gamma (q)}\int_{0}^{\sigma }u^{\left(p-1\right)}\;{\left(\sigma -u\right)}^{q-1}\;{ϒ}_{2}\left(u,\Delta^{*}\left(u\right),{ℵ}^{*}\left(u\right),\Theta^{*}\left(u\right)\right)du. \end{array}$$

Take

$$\begin{array}{ccc} ||ℵ\left(\sigma \right)-{ℵ}^{*}\left(\sigma \right)\left|\right| & = & \left|\right|\frac{p}{\Gamma (q)}\int_{0}^{\sigma }u^{\left(p-1\right)}\;{\left(\sigma -u\right)}^{q-1}\;({ϒ}_{2}\left(u,\Delta \left(u\right),ℵ\left(u\right),\Theta \left(u\right)\right)\\ & - & {ϒ}_{2}\left(u,\Delta^{*}\left(u\right),{ℵ}^{*}\left(u\right),\Theta^{*}\left(u\right)\right))du||\\ & \leq & \frac{p}{\Gamma (q)}\int_{0}^{\sigma }u^{\left(p-1\right)}\;{\left(\sigma -u\right)}^{q-1}\;\left|\right|{ϒ}_{2}\left(u,\Delta \left(u\right),ℵ\left(u\right),\Theta \left(u\right)\right)\\ & - & {ϒ}_{2}\left(u,\Delta^{*}\left(u\right),{ℵ}^{*}\left(u\right),\Theta^{*}\left(u\right)\right)\left|\right|du, \end{array}$$

since Υ_2_ is considered with respect to ℵ and ℵ* so by using previous result and definition of Beta function, we have

$$\begin{array}{ccc} ||ℵ\left(\sigma \right)-{ℵ}^{*}\left(\sigma \right)\left|\right| & \leq & \frac{p}{\Gamma (q)}\int_{0}^{\sigma }u^{\left(p-1\right)}\;{\left(\sigma -u\right)}^{q-1}\;||{ϒ}_{2}\left(ℵ\right)-{ϒ}_{2}\left({ℵ}^{*}\right)\left|\right|du\\ & \leq & \frac{p}{\Gamma (q)}\;T^{\left(p+q-1\right)}\;\beta \left(p,q\right)\;||{ϒ}_{2}\left(ℵ\right)-{ϒ}_{2}\left({ℵ}^{*}\right)\left|\right|\\ & \leq & \frac{p\;T^{\left(p+q-1\right)}\;\Gamma \left(p\right)}{\Gamma (p+q)}\;w_{2}||ℵ\left(\sigma \right)-{ℵ}^{*}\left(\sigma \right)\left|\right|. \end{array}$$

Hence, we obtain

$$||ℵ\left(\sigma \right)-{ℵ}^{*}\left(\sigma \right)||\leq \lambda \;w_{2}\;||ℵ\left(\sigma \right)-{ℵ}^{*}\left(\sigma \right)\left|\right|.$$

This implies that

$$\left(1-\lambda \;w_{2}\right)\;||ℵ\left(\sigma \right)-{ℵ}^{*}\left(\sigma \right)\left|\right|\leq 0.$$

As λ*w*_2_ < 1, this is possible when ||ℵ(*σ*) − ℵ*(*σ*)|| = 0. Thus, ℵ(*σ*) = ℵ*(*σ*). Also, we have

$$\begin{array}{ccc} \Theta (\sigma ) & = & \Theta \left(0\right)+\frac{p}{\Gamma (q)}\int_{0}^{\sigma }u^{\left(p-1\right)}\;{\left(\sigma -u\right)}^{q-1}\;{ϒ}_{3}\left(u,\Delta \left(u\right),ℵ\left(u\right),\Theta \left(u\right)\right)du,\\ \Theta^{*}\left(\sigma \right) & = & \Theta \left(0\right)+\frac{p}{\Gamma (q)}\int_{0}^{\sigma }u^{\left(p-1\right)}\;{\left(\sigma -u\right)}^{q-1}\;{ϒ}_{3}\left(u,\Delta^{*}\left(u\right),{ℵ}^{*}\left(u\right),\Theta^{*}\left(u\right)\right)du. \end{array}$$

Take

$$\begin{array}{ccc} ||\Theta \left(\sigma \right)-\Theta^{*}\left(\sigma \right)\left|\right| & = & \left|\right|\frac{p}{\Gamma (q)}\int_{0}^{\sigma }u^{\left(p-1\right)}\;{\left(\sigma -u\right)}^{q-1}\;({ϒ}_{3}\left(u,\Delta \left(u\right),ℵ\left(u\right),\Theta \left(u\right)\right)\\ & - & {ϒ}_{3}\left(u,\Delta^{*}\left(u\right),{ℵ}^{*}\left(u\right),\Theta^{*}\left(u\right)\right))du||\\ & \leq & \frac{p}{\Gamma (q)}\int_{0}^{\sigma }u^{\left(p-1\right)}\;{\left(\sigma -u\right)}^{q-1}\;\left|\right|{ϒ}_{3}\left(u,\Delta \left(u\right),ℵ\left(u\right),\Theta \left(u\right)\right)\\ & - & {ϒ}_{3}\left(u,\Delta^{*}\left(u\right),{ℵ}^{*}\left(u\right),\Theta^{*}\left(u\right)\right)\left|\right|du, \end{array}$$

since Υ_3_ is considered w.r.t Θ and Θ* so by using previous result and definition of Beta function, we have

$$\begin{array}{ccc} ||\Theta \left(\sigma \right)-\Theta^{*}\left(\sigma \right)\left|\right| & \leq & \frac{p}{\Gamma (q)}\int_{0}^{\sigma }u^{\left(p-1\right)}\;{\left(\sigma -u\right)}^{q-1}\;||{ϒ}_{3}\left(\Theta \right)-{ϒ}_{3}\left(\Theta^{*}\right)\left|\right|du\\ & \leq & \frac{p}{\Gamma (q)}\;T^{\left(p+q-1\right)}\;\beta \left(p,q\right)\;||{ϒ}_{3}\left(\Theta \right)-{ϒ}_{3}\left(\Theta^{*}\right)\left|\right|\\ & \leq & \frac{p\;T^{\left(p+q-1\right)}\;\Gamma \left(p\right)}{\Gamma (p+q)}\;w_{3}||\Theta \left(\sigma \right)-\Theta^{*}\left(\sigma \right)\left|\right|. \end{array}$$

Hence, ||Θ(*σ*) − Θ*(*σ*)|| ≤ λ*w*_3_||Θ(*σ*) − Θ*(*σ*)|| ⇒ (1 − λ*w*_3_)||Θ(*σ*) − Θ*(*σ*)|| ≤ 0.

As λ*w*_3_ < 1, this is possible when ||Θ(*σ*) − Θ*(*σ*)|| = 0. Thus Θ(*σ*) = Θ*(*σ*). That is, (Δ(*σ*), ℵ(*σ*), Θ(*σ*)) = (Δ*(*σ*), ℵ*(*σ*), Θ*(*σ*)). Hence, solution is unique.

## 7 Stability

In this section, we check stability of solution. We use Ulam–Hyers and Ulam–Hayers–Rassias theorems to check it. First, we define the following theorems for our model.

**Definition 7.1**. *Model* (4.1) *is Ulam-Hyers stable* [35], *if for all ϵ*_*i*_ > 0, *there exist* $M_{i}>0\in R$, *which depend on* Υ_*i*_, *i* = 1, 2, 3 *respectively, and for all* (Δ*, ℵ*, Θ*) *satisfying the inequalities*

$$\begin{array}{c} \begin{array}{c} {|}^{FFP}D_{0,\sigma }^{q,p}\Delta^{*}\left(\sigma \right)-{ϒ}_{1}\left(\sigma ,\Delta^{*}\left(\sigma \right),{ℵ}^{*}\left(\sigma \right),\Theta^{*}\left(\sigma \right)\right)|\;\leq \;\epsilon_{1},\\ {|}^{FFP}D_{0,\sigma }^{q,p}{ℵ}^{*}\left(\sigma \right)-{ϒ}_{2}\left(\sigma ,\Delta^{*}\left(\sigma \right),{ℵ}^{*}\left(\sigma \right),\Theta^{*}\left(\sigma \right)\right)|\;\leq \;\epsilon_{2},\\ {|}^{FFP}D_{0,\sigma }^{q,p}\Theta^{*}\left(\sigma \right)-{ϒ}_{3}\left(\sigma ,\Delta^{*}\left(\sigma \right),{ℵ}^{*}\left(\sigma \right),\Theta^{*}\left(\sigma \right)\right)|\;\leq \;\epsilon_{3}, \end{array} \end{array}$$ (7.1)

*there exists* (Δ, ℵ, Θ) ∈ Ξ *satisfying model* (4.1) *with the condition*

$$\begin{array}{c} \begin{array}{c} |\Delta^{*}\left(\sigma \right)-\Delta \left(\sigma \right)|\;\leq \;M_{1}\;\epsilon_{1},\\ |{ℵ}^{*}\left(\sigma \right)-ℵ\left(\sigma \right)|\;\leq \;M_{2}\;\epsilon_{2},\\ |\Theta^{*}\left(\sigma \right)-\Theta \left(\sigma \right)|\;\leq \;M_{3}\;\epsilon_{3}. \end{array} \end{array}$$ (7.2)

**Remark 7.2**. (Δ*, ℵ*, Θ*) ∈ Ξ *is a solution of model* (7.1) *iff* ∃ *η*_*i*_ ∈ *C*([0, *T*], [0, ∞)) *such that for all σ* ∈ *J*,

*(i)* |*η*_*i*_(*σ*)| < *ϵ*_*i*_,
*(ii)*
DFFP0,σq,pΔ*(σ)=ϒ1(σ,Δ*(σ),ℵ*(σ),Θ*(σ))+η1(σ),DFFP0,σq,pℵ*(σ)=ϒ2(σ,Δ*(σ),ℵ*(σ),Θ*(σ))+η2(σ),DFFP0,σq,pΘ*(σ)=ϒ3(σ,Δ*(σ),ℵ*(σ),Θ*(σ))+η3(σ). (7.3)

**Theorem 7.3**. *The fractal fraction model is Ulam–Hayers stable on J such that* λ*w*_*i*_ < 1, *where w*_*i*_ *and* λ *are defined as above*.

*Proof*. Let *ϵ*_1_ > 0 and $\Delta^{*}\in Y$ s.t

$${|}^{FFP}D_{0,\sigma }^{q,p}\Delta^{*}\left(\sigma \right)-{ϒ}_{1}\left(\sigma ,\Delta^{*}\left(\sigma \right),{ℵ}^{*}\left(\sigma \right),\Theta^{*}\left(\sigma \right)\right)|\;\leq \;\epsilon_{1},$$

by above remark, we have

$$\begin{array}{ccc} \Delta^{*}\left(\sigma \right) & = & \Delta \left(0\right)+\frac{p}{\Gamma (q)}\int_{0}^{\sigma }u^{\left(p-1\right)}\;{\left(\sigma -u\right)}^{q-1}\;{ϒ}_{1}\left(u,\Delta^{*}\left(u\right),{ℵ}^{*}\left(u\right),\Theta^{*}\left(u\right)\right)du\\ & + & \frac{p}{\Gamma (q)}\int_{0}^{\sigma }u^{\left(p-1\right)}\;{\left(\sigma -u\right)}^{q-1}\;\eta_{1}\left(u\right)\;du. \end{array}$$ (7.4)

As Δ∈Y is the unique solution, then

$$\Delta \left(\sigma \right)=\Delta \left(0\right)+\frac{p}{\Gamma (q)}\int_{0}^{\sigma }u^{\left(p-1\right)}\;{\left(\sigma -u\right)}^{q-1}\;{ϒ}_{1}\left(u,\Delta \left(u\right),ℵ\left(u\right),\Theta \left(u\right)\right)du.$$

That is

$$\begin{array}{ccc} |\Delta^{*}\left(\sigma \right)-\Delta \left(\sigma \right)|\\ & = & |\frac{p}{\Gamma (q)}\int_{0}^{\sigma }u^{\left(p-1\right)}\;{\left(\sigma -u\right)}^{q-1}\;\eta_{1}\left(u\right)\;du\\ & + & \frac{p}{\Gamma (q)}\int_{0}^{\sigma }u^{\left(p-1\right)}\;{\left(\sigma -u\right)}^{q-1}[{ϒ}_{1}\left(u,\Delta^{*}\left(u\right),{ℵ}^{*}\left(u\right),\Theta^{*}\left(u\right)\right)\\ & - & {ϒ}_{1}\left(u,\Delta \left(u\right),ℵ\left(u\right),\Theta \left(u\right)\right)\;]\;du|\\ & \leq & \frac{p}{\Gamma (q)}\int_{0}^{\sigma }u^{\left(p-1\right)}\;{\left(\sigma -u\right)}^{q-1}\;\left|\eta_{1}\left(u\right)\right|du\\ & + & \frac{p}{\Gamma (q)}\int_{0}^{\sigma }u^{\left(p-1\right)}\;{\left(\sigma -u\right)}^{q-1}\left|\right|{ϒ}_{1}\left(u,\Delta \left(u\right),ℵ\left(u\right),\Theta \left(u\right)\right)\\ & - & {ϒ}_{1}\left(u,\Delta^{*}\left(u\right),{ℵ}^{*}\left(u\right),\Theta^{*}\left(u\right)\right)\;\left|\right|\;du\\ & \leq & \frac{p}{\Gamma (q)}\;\beta \left(p,q\right)\;T^{\left(p+q-1\right)}\;\left|\eta_{1}\left(u\right)\right|\\ & + & \frac{p}{\Gamma (q)}\;\beta \left(p,q\right)\;T^{\left(p+q-1\right)}\;w_{1}\;||\Delta^{*}-\Delta \left|\right|\\ & \leq & \frac{p}{\Gamma (q)}\;\beta \left(p,q\right)\;T^{\left(p+q-1\right)}\;\epsilon_{1}\\ & + & \frac{p}{\Gamma (q)}\;\beta \left(p,q\right)\;T^{\left(p+q-1\right)}\;w_{1}\;||\Delta^{*}-\Delta \left|\right|\\ & \leq & \lambda \;\epsilon_{1}+\lambda \;w_{1}\;||\Delta^{*}-\Delta \left|\right|. \end{array}$$

Hence, we have

$$\begin{array}{ccc} \left|\right|\Delta^{*}-\Delta \left|\right|\; & \leq & \;\lambda \;\epsilon_{1}+\;\lambda \;w_{1}\;||\Delta^{*}-\Delta \left|\right|\\ \left(1-\lambda \;w_{1}\right)\;||\Delta^{*}-\Delta \left|\right|\; & \leq & \;\lambda \;\epsilon_{1}\\ \left|\right|\Delta^{*}-\Delta \left|\right|\; & \leq & \;\frac{\lambda \;\epsilon_{1}}{\left(1-\lambda \;w_{1}\right)}. \end{array}$$

If $\frac{\lambda }{\left(1-\lambda \;w_{1}\right)}=M_{1}$, then ||Δ* − Δ|| ≤ *M*_1_ *ϵ*_1_.

Similarly, we can prove that ||ℵ* − ℵ|| ≤ *M*_2_ *ϵ*_2_, and ||Θ* − Θ|| ≤ *M*_3_ *ϵ*_3_.

Thus Ulam–Hayers stability criteria is fulfilled by our fractal–fractional model.

**Definition 7.4**. *We define the Ulam–Hayers–Rassias stability criteria for our fractal–fractional model* ([36]). *Model* (4.1) *is Ulam-Hyers–Rassias stable with respect to the functions ψ*_*i*_, *if for all ϵ*_*i*_ > 0, *there exist M*_*i*_ > 0 ∈ [0, ∞), *which depend on* Υ_*i*_ *and ψ*_*i*_, *i* = 1, 2, 3 *respectively and for all* (Δ*, ℵ*, Θ*) *satisfying the inequalities*:

$$\begin{array}{c} \begin{array}{c} {|}^{FFP}D_{0,\sigma }^{q,p}\Delta^{*}\left(\sigma \right)-{ϒ}_{1}\left(\sigma ,\Delta^{*}\left(\sigma \right),{ℵ}^{*}\left(\sigma \right),\Theta^{*}\left(\sigma \right)\right)|\;\leq \;\epsilon_{1}\;\psi_{1}\left(\sigma \right),\\ {|}^{FFP}D_{0,\sigma }^{q,p}{ℵ}^{*}\left(\sigma \right)-{ϒ}_{2}\left(\sigma ,\Delta^{*}\left(\sigma \right),{ℵ}^{*}\left(\sigma \right),\Theta^{*}\left(\sigma \right)\right)|\;\leq \;\epsilon_{2}\;\psi_{2}\left(\sigma \right),\\ {|}^{FFP}D_{0,\sigma }^{q,p}\Theta^{*}\left(\sigma \right)-{ϒ}_{3}\left(\sigma ,\Delta^{*}\left(\sigma \right),{ℵ}^{*}\left(\sigma \right),\Theta^{*}\left(\sigma \right)\right)|\;\leq \;\epsilon_{3}\;\psi_{3}\left(\sigma \right), \end{array} \end{array}$$ (7.5)

*there exists* (Δ, ℵ, Θ) ∈ Ξ *satisfying model* (4.1) *with the conditions*:

$$\begin{array}{c} \begin{array}{c} |\Delta^{*}\left(\sigma \right)-\Delta \left(\sigma \right)|\;\leq \;M_{1}\;\epsilon_{1}\;\psi_{1}\left(\sigma \right),\\ |{ℵ}^{*}\left(\sigma \right)-ℵ\left(\sigma \right)|\;\leq \;M_{2}\;\epsilon_{2}\;\psi_{2}\left(\sigma \right),\\ |\Theta^{*}\left(\sigma \right)-\Theta \left(\sigma \right)|\;\leq \;M_{3}\;\epsilon_{3}\;\psi_{3}\left(\sigma \right). \end{array} \end{array}$$ (7.6)

**Remark 7.5**. (Δ*, ℵ*, Θ*) ∈ Ξ *is a solution iff* ∃ *η*_*i*_ ∈ *C*(*J*, [0, ∞)) *such that for all σ* ∈ *J*,

*(i)* |*η*_*i*_(*σ*)| < *ϵ*_*i*_ *ψ*_*i*_(*σ*),
*(ii)*
DFFP0,σq,pΔ*(σ)=ϒ1(σ,Δ*(σ),ℵ*(σ),Θ*(σ))+η1(σ),DFFP0,σq,pℵ*(σ)=ϒ2(σ,Δ*(σ),ℵ*(σ),Θ*(σ))+η2(σ),DFFP0,σq,pΘ*(σ)=ϒ3(σ,Δ*(σ),ℵ*(σ),Θ*(σ))+η3(σ). (7.7)

**Theorem 7.6**. *The fractal–fractional model* (4.1) *is Ulam–Hayers–Rassias stable when the following conditions are satisfied for all σ* ∈ *J there exists nondecreasing mappings ψ*_*i*_ ∈ *C*(*J*, [0, ∞)) *and* *ξ*_*i*_ > 0 *depending upon ψ*_*i*_ *such that* IFFP0,σq,pψi(σ)<ξi(ψi)ψi(σ).

*Proof*. Let *ϵ*_1_ > 0 and $\Delta^{*}\in Y$ such that

$${|}^{FFP}D_{0,\sigma }^{q,p}\Delta^{*}\left(\sigma \right)-{ϒ}_{1}\left(\sigma ,\Delta^{*}\left(\sigma \right),{ℵ}^{*}\left(\sigma \right),\Theta^{*}\left(\sigma \right)\right)|\;\leq \;\epsilon_{1}\;\psi_{1}\left(\sigma \right),$$

then, by the conditions of remark 7.5, we consider

$$\begin{array}{cc} \Delta^{*}\left(\sigma \right) & =\Delta \left(0\right)+\frac{p}{\Gamma (q)}\int_{0}^{\sigma }u^{\left(p-1\right)}\;{\left(\sigma -u\right)}^{q-1}\;{ϒ}_{1}\left(u,\Delta^{*}\left(u\right),{ℵ}^{*}\left(u\right),\Theta^{*}\left(u\right)\right)du\\ & +\frac{p}{\Gamma (q)}\int_{0}^{\sigma }u^{\left(p-1\right)}\;{\left(\sigma -u\right)}^{q-1}\;\eta_{1}\left(u\right)\;du. \end{array}$$

As Δ∈Y is the unique solution, then

$$\Delta \left(\sigma \right)=\Delta \left(0\right)+\frac{p}{\Gamma (q)}\int_{0}^{\sigma }u^{\left(p-1\right)}\;{\left(\sigma -u\right)}^{q-1}\;{ϒ}_{1}\left(u,\Delta \left(u\right),ℵ\left(u\right),\Theta \left(u\right)\right)du.$$

Therefore, we get

$$\begin{array}{ccc} |\Delta^{*}\left(\sigma \right)-\Delta \left(\sigma \right)|\\ & = & |\frac{p}{\Gamma (q)}\int_{0}^{\sigma }u^{\left(p-1\right)}\;{\left(\sigma -u\right)}^{q-1}\;\eta_{1}\left(u\right)\;du\\ & + & \frac{p}{\Gamma (q)}\int_{0}^{\sigma }u^{\left(p-1\right)}\;{\left(\sigma -u\right)}^{q-1}\\ & [ & {ϒ}_{1}\left(u,\Delta^{*}\left(u\right),{ℵ}^{*}\left(u\right),\Theta^{*}\left(u\right)\right)-{ϒ}_{1}\left(u,\Delta \left(u\right),ℵ\left(u\right),\Theta \left(u\right)\right)\;]\;du|\\ & \leq & \frac{p}{\Gamma (q)}\int_{0}^{\sigma }u^{\left(p-1\right)}\;{\left(\sigma -u\right)}^{q-1}\;\left|\eta_{1}\left(u\right)\right|du\\ & + & \frac{p}{\Gamma (q)}\int_{0}^{\sigma }u^{\left(p-1\right)}\;{\left(\sigma -u\right)}^{q-1}\\ & \left|\right| & {ϒ}_{1}\left(u,\Delta \left(u\right),ℵ\left(u\right),\Theta \left(u\right)\right)-{ϒ}_{1}\left(u,\Delta^{*}\left(u\right),{ℵ}^{*}\left(u\right),\Theta^{*}\left(u\right)\right)\;\left|\right|\;du\\ & \leq & \frac{p}{\Gamma (q)}\int_{0}^{\sigma }u^{\left(p-1\right)}\;{\left(\sigma -u\right)}^{q-1}\;\epsilon_{1}\;\psi_{1}\left(u\right)\;du\\ & + & \frac{p}{\Gamma (q)}\;\beta \left(p,q\right)\;T^{\left(p+q-1\right)}\;w_{1}\;||\Delta^{*}-\Delta \left|\right|\\ & \leq & \epsilon_{1}\;\xi_{1}\left(\psi_{i}\right)\;\psi_{1}\left(\sigma \right)+\lambda \;w_{1}\;||\Delta^{*}-\Delta \left|\right|. \end{array}$$

Hence, we have

$$\begin{array}{ccc} \left|\right|\Delta^{*}-\Delta \left|\right|\; & \leq & \;\epsilon_{1}\;\xi_{1}\left(\psi_{1}\right)\;\psi_{1}\left(\sigma \right)+\;\lambda \;w_{1}\;||\Delta^{*}-\Delta \left|\right|\\ \left(1-\lambda \;w_{1}\right)\;||\Delta^{*}-\Delta \left|\right|\; & \leq & \;\epsilon_{1}\;\xi_{1}\left(\psi_{1}\right)\;\psi_{1}\left(\sigma \right)\\ \left|\right|\Delta^{*}-\Delta \left|\right|\; & \leq & \;\frac{\epsilon_{1}\;\xi_{1}\left(\psi_{1}\right)\;\psi_{1}\left(\sigma \right)}{\left(1-\lambda \;w_{1}\right)}. \end{array}$$

If $\frac{\xi_{1}\left(\psi_{1}\right)}{\left(1-\lambda \;w_{1}\right)}=M_{1}\left({ϒ}_{1},\psi_{1}\right)$ then, we get

$$\left|\right|\Delta^{*}-\Delta \left|\right|\;\leq \;\epsilon_{1}\;\psi_{1}\left(\sigma \right)M_{1}\left({ϒ}_{1},\psi_{1}\right).$$

Similarly, we can prove that

$$\left|\right|{ℵ}^{*}-ℵ\left|\right|\;\leq \;\epsilon_{2}\;\psi_{2}\left(\sigma \right)M_{2}\left({ϒ}_{2},\psi_{2}\right),$$

$$\left|\right|\Theta^{*}-\Theta \left|\right|\;\leq \;\epsilon_{3}\;\psi_{3}\left(\sigma \right)M_{3}\left({ϒ}_{3},\psi_{3}\right).$$

Thus, Ulam–Hayers–Rassias stability criteria is fulfilled by our fractal–fractional model.

## 8 Numerical algorithm

Now, we make a numerical scheme using the two–point Lagrangian interpolation formula for our fractal–fractional model as in [37]. The difference between our scheme and others is that in our model Υ_1_ and Υ_3_ depend on *σ* and (*σ* − *τ*) so we deal it differently in the end.

First we take *σ* = *σ*_*n*+1_ and $u^{p-1}\;{ϒ}_{i}\left(u,S\left(u\right),ℵ\left(u\right),\Theta \left(u\right)\right)={Ϝ}_{i}\left(u\right)$ and we get

$$\begin{array}{c} \begin{array}{c} \Delta \left(\sigma_{n+1}\right)=\Delta \left(0\right)+\frac{p}{\Gamma (q)}\int_{0}^{\sigma_{n+1}}{\left(\sigma_{n+1}-u\right)}^{q-1}\;{Ϝ}_{1}\left(u\right)du,\\ ℵ\left(\sigma_{n+1}\right)=ℵ\left(0\right)+\frac{p}{\Gamma (q)}\int_{0}^{\sigma_{n+1}}{\left(\sigma_{n+1}-u\right)}^{q-1}\;{Ϝ}_{2}\left(u\right)du,\\ \Theta \left(\sigma_{n+1}\right)=\Theta \left(0\right)+\frac{p}{\Gamma (q)}\int_{0}^{\sigma_{n+1}}{\left(\sigma_{n+1}-u\right)}^{q-1}\;{Ϝ}_{3}\left(u\right)du. \end{array} \end{array}$$ (8.1)

Approximating integral as the sum of integrals on sub intervals, we have

$$\begin{array}{c} \begin{array}{c} \Delta \left(\sigma_{n+1}\right)=\Delta_{0}+\frac{p}{\Gamma (q)}\sum_{j=0}^{n}\int_{\sigma_{j}}^{\sigma_{j+1}}{\left(\sigma_{n+1}-u\right)}^{q-1}{Ϝ}_{1}\left(u\right)du,\\ ℵ\left(\sigma_{n+1}\right)={ℵ}_{0}+\frac{p}{\Gamma (q)}\sum_{j=0}^{n}\int_{\sigma_{j}}^{\sigma_{j+1}}{\left(\sigma_{n+1}-u\right)}^{q-1}{Ϝ}_{2}\left(u\right)du,\\ \Theta \left(\sigma_{n+1}\right)=\Theta_{0}+\frac{p}{\Gamma (q)}\sum_{j=0}^{n}\int_{\sigma_{j}}^{\sigma_{j+1}}{\left(\sigma_{n+1}-u\right)}^{q-1}{Ϝ}_{3}\left(u\right)du. \end{array} \end{array}$$ (8.2)

Now, we approximate the functions ${Ϝ}_{i}\left(u\right)$ by two points Lagrange interpolation polynomials on the interval [*σ*_*j*_, *σ*_*j*+1_]. We can write

$$\begin{array}{ccc} {Ϝ}_{1}^{*}\left(u\right) & = & \frac{u-\sigma_{j-1}}{\sigma_{j}-\sigma_{j-1}}\;\sigma_{j}^{p-1}\;{ϒ}_{1}\left(u_{j},\Delta_{j}\left(u\right),{ℵ}_{j}\left(u\right),\Theta_{j}\left(u\right)\right)\\ & - & \frac{u-\sigma_{j}}{\sigma_{j}-\sigma_{j-1}}\;\sigma_{j-1}^{p-1}\;{ϒ}_{1}(u_{j-1},\Delta_{j-1}\left(u\right),{ℵ}_{j-1}\left(u\right),\Theta_{j-1}\left(u\right),\\ {Ϝ}_{2}^{*}\left(u\right) & = & \frac{u-\sigma_{j-1}}{\sigma_{j}-\sigma_{j-1}}\;\sigma_{j}^{p-1}\;{ϒ}_{2}\left(u_{j},\Delta_{j}\left(u\right),{ℵ}_{j}\left(u\right),\Theta_{j}\left(u\right)\right)\\ & - & \frac{u-\sigma_{j}}{\sigma_{j}-\sigma_{j-1}}\;\sigma_{j-1}^{p-1}\;{ϒ}_{2}(u_{j-1},\Delta_{j-1}\left(u\right),{ℵ}_{j-1}\left(u\right),\Theta_{j-1}\left(u\right),\\ {Ϝ}_{3}^{*}\left(u\right) & = & \frac{u-\sigma_{j-1}}{\sigma_{j}-\sigma_{j-1}}\;\sigma_{j}^{p-1}\;{ϒ}_{3}\left(u_{j},\Delta_{j}\left(u\right),{ℵ}_{j}\left(u\right),\Theta_{j}\left(u\right)\right)\\ & - & \frac{u-\sigma_{j}}{\sigma_{j}-\sigma_{j-1}}\;\sigma_{j-1}^{p-1}\;{ϒ}_{3}(u_{j-1},\Delta_{j-1}\left(u\right),{ℵ}_{j-1}\left(u\right),\Theta_{j-1}\left(u\right). \end{array}$$

Thus, we have

$$\begin{array}{c} \begin{array}{c} \Delta \left(\sigma_{n+1}\right)=\Delta_{0}+\frac{p}{\Gamma (q)}\sum_{j=0}^{n}\int_{\sigma_{j}}^{\sigma_{j+1}}{\left(\sigma_{n+1}-u\right)}^{q-1}{Ϝ}_{1}^{*}\left(u\right)du,\\ ℵ\left(\sigma_{n+1}\right)={ℵ}_{0}+\frac{p}{\Gamma (q)}\sum_{j=0}^{n}\int_{\sigma_{j}}^{\sigma_{j+1}}{\left(\sigma_{n+1}-u\right)}^{q-1}{Ϝ}_{2}^{*}\left(u\right)du,\\ \Theta \left(\sigma_{n+1}\right)=\Theta_{0}+\frac{p}{\Gamma (q)}\sum_{j=0}^{n}\int_{\sigma_{j}}^{\sigma_{j+1}}{\left(\sigma_{n+1}-u\right)}^{q-1}{Ϝ}_{3}^{*}\left(u\right)du. \end{array} \end{array}$$ (8.3)

Using values of ${Ϝ}_{i}^{*}\left(u\right)$ we integrate the above integrals according to limits and taking *σ*_*j*_ − *σ*_*j*−1_ = *h*, we get the final results.

$$\begin{array}{ccc} \Delta (n+1) & = & \Delta_{0}+\frac{p\;h^{q}}{\Gamma (q+2)}\sum_{j=0}^{n}[\sigma_{j}^{p-1}{ϒ}_{1}\left(u_{j},\Delta_{j},{ℵ}_{j},\Theta_{j}\right)Z_{1}\\ & - & \sigma_{j-1}^{p-1}\;{ϒ}_{1}\left(u_{j-1},\Delta_{j-1},{ℵ}_{j-1},\Theta_{j-1}\right)Z_{2}],\\ ℵ(n+1) & = & {ℵ}_{0}+\frac{p\;h^{q}}{\Gamma (q+2)}\sum_{j=0}^{n}[\sigma_{j}^{p-1}{ϒ}_{2}\left(u_{j},\Delta_{j},{ℵ}_{j},\Theta_{j}\right)\;Z_{1}\\ & - & \sigma_{j-1}^{p-1}\;{ϒ}_{2}\left(u_{j-1},\Delta_{j-1},{ℵ}_{j-1},\Theta_{j-1}\right)\;Z_{2}],\\ \Theta (n+1) & = & \Theta_{0}+\frac{p\;h^{q}}{\Gamma (q+2)}\sum_{j=0}^{n}[\sigma_{j}^{p-1}{ϒ}_{3}\left(u_{j},\Delta_{j},{ℵ}_{j},\Theta_{j}\right)\;Z_{1}\\ & - & \sigma_{j-1}^{p-1}\;{ϒ}_{3}\left(u_{j-1},\Delta_{j-1},{ℵ}_{j-1},\Theta_{j-1}\right)\;Z_{2}]. \end{array}$$

where

$Z_{1}={\left(n+1-j\right)}^{q}\;\left(n-j+2+q\right)-{\left(n-j\right)}^{q}\;\left(n-j+2+2q\right)$,

$Z_{2}={\left(n+1-j\right)}^{q+1}-{\left(n-j\right)}^{q}\;\left(n-j+1+q\right)$.

Since in the original model Υ_1_ and Υ_3_, Θ depends on *σ* and (*σ* − *τ*) = *σ*_1_(*say*), so we write Υ_1_ = *U*_1_(*σ*_*j*_, Δ_*j*_, ℵ_*j*_, Θ_*j*_) + *U*_3_(*σ*_1*j*_, Θ_*j*_) and Υ_3_ = *U*_2_(*σ*_*j*_, Δ_*j*_, ℵ_*j*_, Θ_*j*_) − *U*_3_(*σ*_1*j*_, Θ_*j*_) where

*U*_1_(*σ*_*j*_, Δ_*j*_, ℵ_*j*_, Θ_*j*_) = Π*θ* − *β*_0_ *f*(ℵ(*σ*))Δ(*σ*) − (*μ* + *ν*)Δ(*σ*),
*U*_2_(*σ*_*j*_, Δ_*j*_, ℵ_*j*_, Θ_*j*_) = (1 − Π)*θ* + *ν*Δ(*σ*) + *κ*ℵ(*σ*) − *μ*Θ(*σ*),
*U*_3_(*σ*_1*j*_, Θ_*j*_) = *ζ*Θ(*σ* − *τ*).

Hence, our numerical scheme is

$$\begin{array}{c} \begin{array}{cc} \Delta (n+1) & =\Delta_{0}+\frac{p\;h^{q}}{\Gamma (q+2)}\sum_{j=0}^{n}[\sigma_{j}^{p-1}\left(U_{1}\left(\sigma_{j},\Delta_{j},{ℵ}_{j},\Theta_{j}\right)+U_{3}\left(\sigma_{1j},\Theta_{j}\right)\right)\;Z_{1}\\ & -\sigma_{j-1}^{p-1}\;\left(U_{1}\left(\sigma_{j-1},\Delta_{j-1},{ℵ}_{j-1},\Theta_{j-1}\right)+U_{3}\left(\sigma_{1j},\Theta_{j}\right)\right)\;Z_{2}],\\ ℵ(n+1) & ={ℵ}_{0}+\frac{p\;h^{q}}{\Gamma (q+2)}\sum_{j=0}^{n}[\sigma_{j}^{p-1}{ϒ}_{2}\left(u_{j},\Delta_{j},{ℵ}_{j},\Theta_{j}\right)\;Z_{1}\\ & -\sigma_{j-1}^{p-1}\;{ϒ}_{2}\left(u_{j-1},\Delta_{j-1},{ℵ}_{j-1},\Theta_{j-1}\right)\;Z_{2}],\\ \Theta (n+1) & =\Theta_{0}+\frac{p\;h^{q}}{\Gamma (q+2)}\sum_{j=0}^{n}[\sigma_{j}^{p-1}\left(U_{2}\left(\sigma_{j},\Delta_{j},{ℵ}_{j},\Theta_{j}\right)-U_{3}\left(\sigma_{1j},\Theta_{j}\right)\right)\;Z_{1}\\ & -\sigma_{j-1}^{p-1}\;\left(U_{2}\left(\sigma_{j-1},\Delta_{j-1},{ℵ}_{j-1},\Theta_{j-1}\right)-U_{3}\left(\sigma_{1j},\Theta_{j}\right)\right)\;Z_{2}]. \end{array} \end{array}$$

## 9 Discussion through simulations based on numerical algorithm

In this section, we see the simulation of Δ, ℵ and Θ under the effect of several fractal-fractional orders and also the behaviour of Δ, ℵ and Θ model with respect to some parameters and compare the results of fractal-fractional model to the ordinary differential model. We take the parameters as taken for figure 2 in [6], Π = 0.5, *θ* = 0.8, *β*_0_ = 0.02, *μ* = 0.1, *ν* = 0.2, *ζ* = 0.01, *κ* = 0.2, *τ* = 7.3, *α* = 1 and some estimated initial conditions Δ(0) = 3, ℵ(0) = 1, Θ(0) = 0.1.

**Fig 1:**
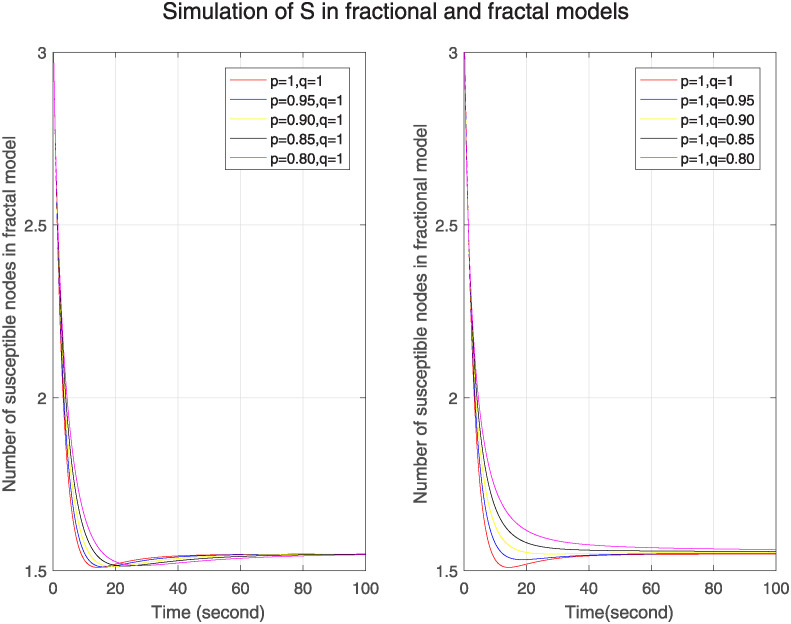
Trajectories of Δ(*σ*) for arbitrary fractal orders p when q=1 and different fractional orders q when p=1.

**Fig 2:**
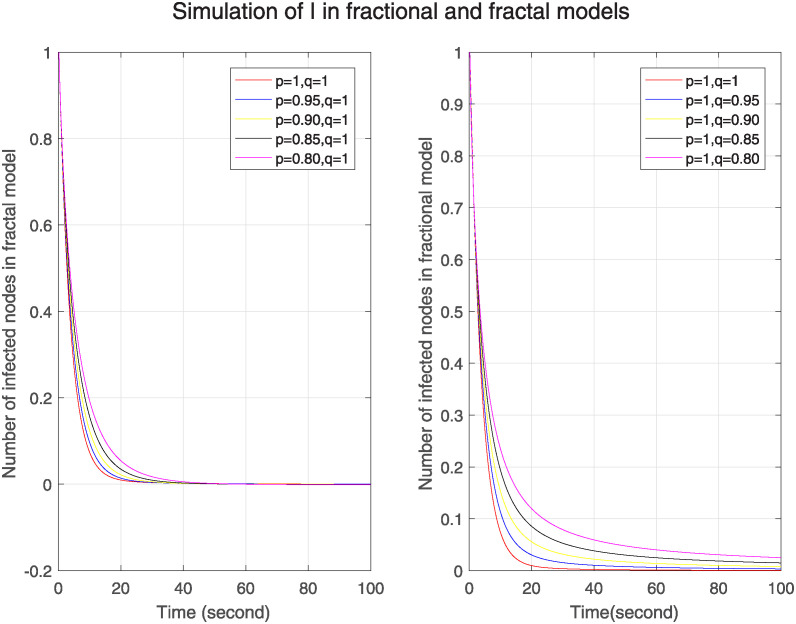
Trajectories of ℵ(*σ*) for arbitrary fractal orders p when q=1 and different fractional orders q when p=1.

**Fig 3:**
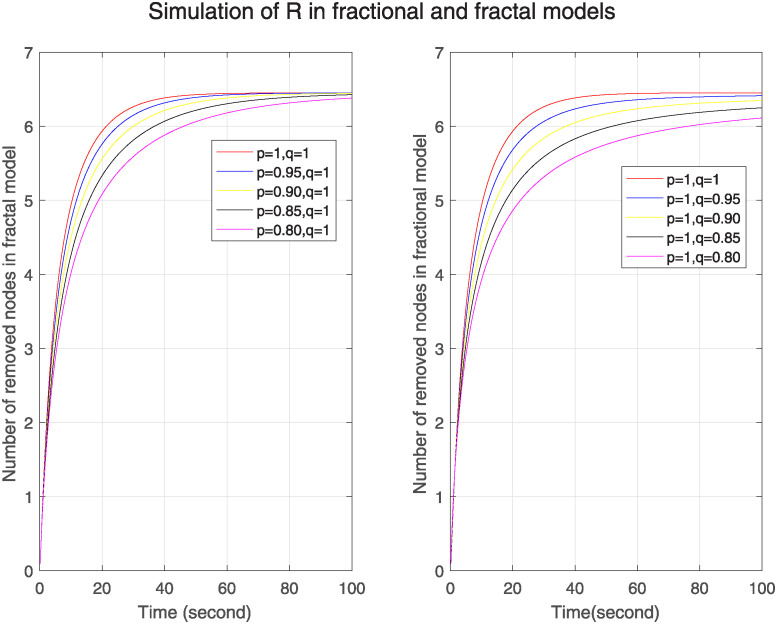
Trajectories of Θ(*σ*) for arbitrary fractal orders p when q=1 and different fractional orders q when p=1.

Figs 1–3 show the simulation of Δ, ℵ and Θ for different fractal and fractional orders separately. We can see in Fig 1, the simulation of Δ in one figure under the different fractional orders keeping fractal order one and in the other figure under different fractal orders by keeping fractional order one. We see that initially number of susceptible nodes is more at lower fractional and fractal orders. In fractal model, after 20s, this number becomes less at lower fractal order and then converges after 80s. On the other hand, in fractional model the no of susceptible nodes is more at lower fractional orders throughout the time period and it converges slowly as compared to fractal model. So, we can see that in fractal model nodes converge more quickly that means no. of susceptible nodes is less affected by previous nodes. So we can predict that it has less memory effect, less sensitivity and more stability. Similarly Fig 2 is for ℵ. It represents higher number of nodes of infected nodes at lower fractal or fractional orders in both models. However, the convergence in fractal model is rapid so we can conclude that it has less memory effect, less sensitivity and more stability. Fig 3 is for Θ. In this figure, in both models the number of removed nodes is less at lower fractional or fractal orders but in fractal model the number of nodes is more than the number of nodes in fractional model at different orders. The nodes become stable more quickly as compared to fractional model. The fractal model has less memory effect, low sensitivity to initial conditions and limited information retention. It shows the uniqueness of the solution and existence of fixed point.

**Fig 4:**
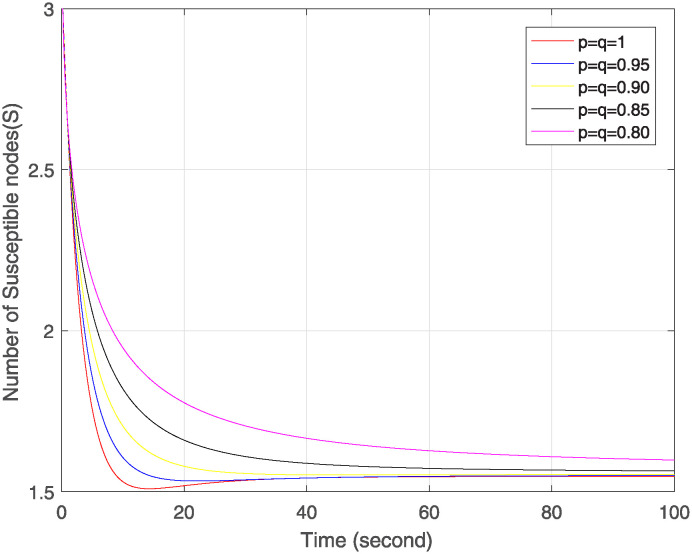
Trajectories of Δ(*σ*) for different orders of p=q.

**Fig 5:**
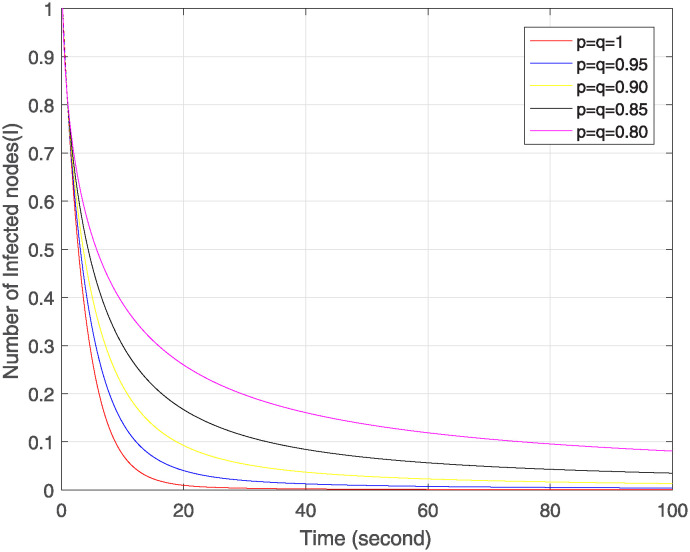
Trajectories of ℵ(*σ*) with different orders of p=q.

**Fig 6:**
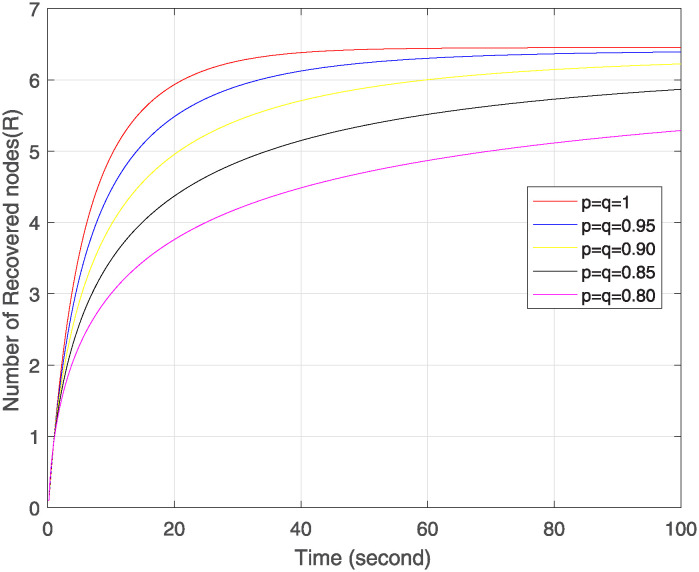
Trajectories of Θ(*σ*) with different orders of p=q.

Figs 4–6 show the simulation of Δ, ℵ and Θ under the combined effect of arbitrary fractal and fractional orders. Fig 4 shows that as the fractal-fractional orders decrease, the number of susceptible nodes is higher at lower fractal-fractional orders and after some time it becomes stable rapidly and converges to the same limit except at order 0.80. This behavior depicts that these nodes have a higher risk of infection or perturbation. They are more sensitive to external influences, have strong memory effect and rapid stabilization. Fig 5 represents the behavior of infected nodes. The number of infected nodes first decreases then becomes stable at all fractal fractional orders. It depicts that the system has resilience to adapt infection where some nodes are still resistant and preventing further infection. Also, the number of nodes becomes zero at fractal fractional level one which describes that infection has been eradicated while at lower level of fractal fraction when no. of nodes goes on decreases but does not become zero show that the system has not been fully eradicated but also has the effect of memory which contributes to the persistence of infected nodes. In Fig 6, we see that number of removed nodes reduces as we reduce the fractal-fractional orders which are represented by p and q. It describes that the memory effect is less in these nodes and these nodes have high stability and fast convergence. It depicts that at lower level of fractal fraction the persistence and containment of infection is high.

**Fig 7:**
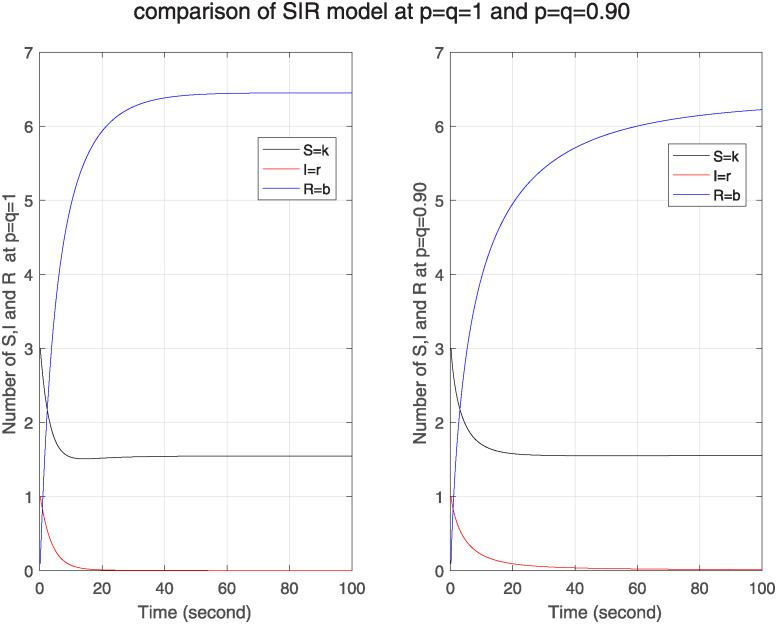
Comparison of Δ, ℵ, and Θ model for classical and FF model at level 0.90.

Moreover in Fig 7, we compare Δ, ℵ and Θ model for fractal-fractional orders p, q as p=q=1 and p=q=0.90 which shows behaviour of three nodes in one figure. We can see from the comparison of nodes that at lower level of fractal fractional, memory effect is stronger and has more influence of initial conditions and previous nodes.

**Fig 8:**
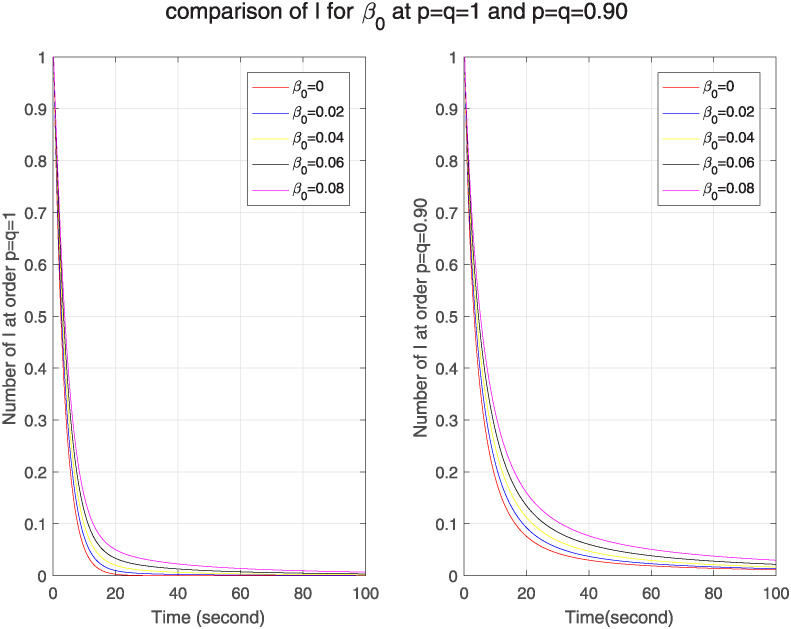
The effect of varying initial infection rate *β*_0_ on infected nodes for classical and FF model at level 0.90.

Since in our original model, infection rate depends on initial infection rate *β*_0_ and also on a function of ℵ which depends on another variable called *α*. Therefore we see the impact of both variables on our fractal-fractional model too. Fig 8, first we see that in both models no. of infected nodes goes on increasing for higher initial infection rate i.e. as initial infection rate increases, no. of infected nodes also increases. It describes that outbreak is escalating and infected nodes are increasing rapidly. On the other hand, when we compare both models it shows that at lower fractal fractional order the malware spreads faster and has stronger memory effect than the integer order model.

**Fig 9:**
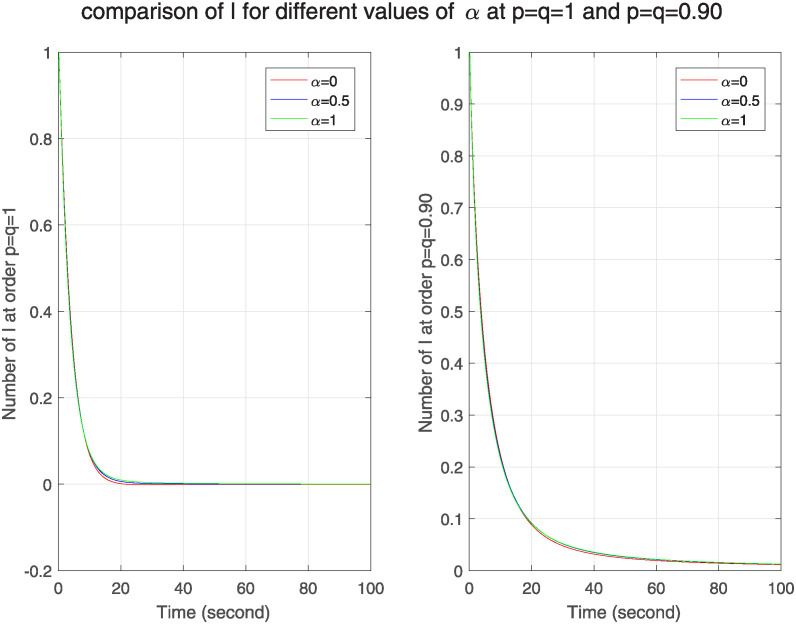
The effect of varying variable to adjust the infection rate sensitivity *α* on infected nodes for classical and FF model at level 0.90.

Also in the original model, *α* is used to adjust the infection rate sensitivity to ℵ. Here *α* = 0 means constant infection rate. According to the authors in [6], at *α* = 1, the scale of malware spreading is smaller than the rate at *α* = 0. From Fig 9 first we see that this condition is satisfied in both models. Also we observe that no. of infected nodes goes on decreasing more quickly as time passes in FF model as compared to integer order model. As a result, we can see that FF model has weaker memory effect and more stability and resilience to outbreaks in the nodes.

**Fig 10:**
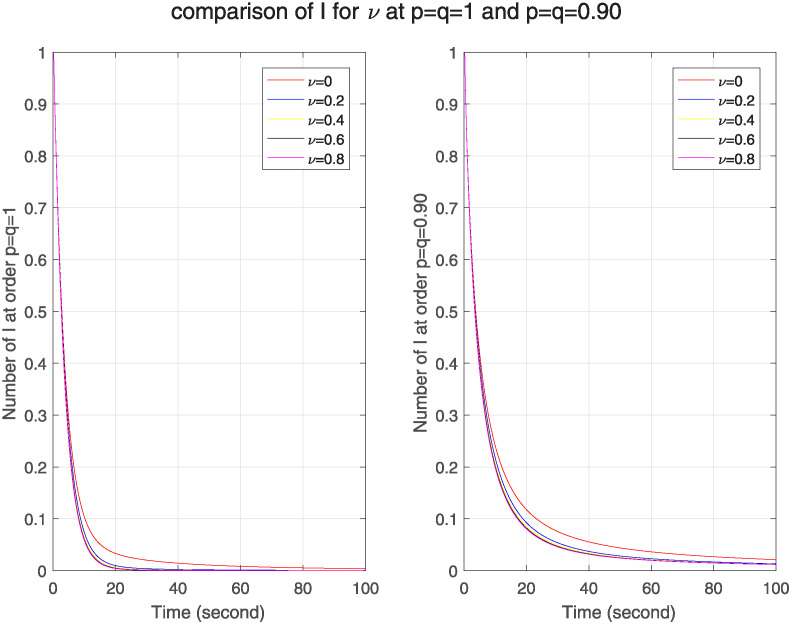
The effect of varying real-time immune rate *ν* on infected nodes for classical and FF model at level 0.90.

**Fig 11:**
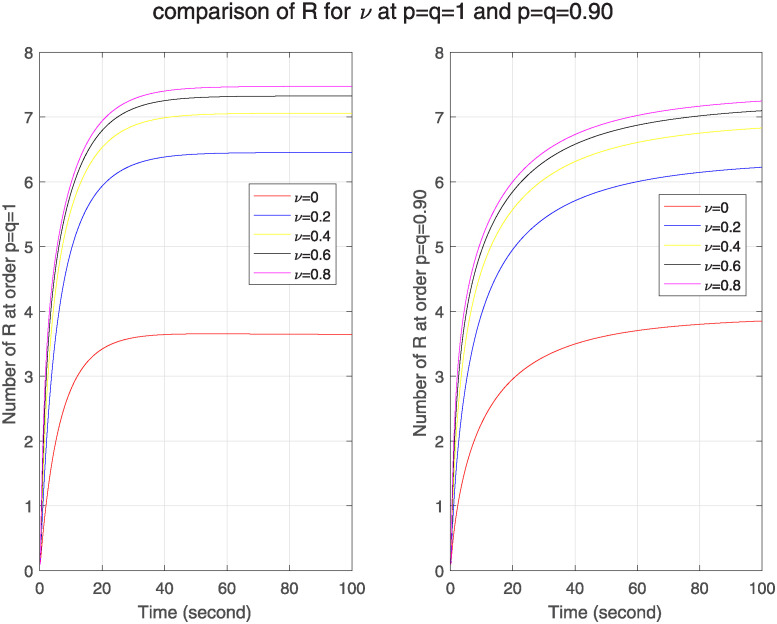
The effect of varying real-time immune rate *ν* on removed nodes for classical and FF model at level 0.90.

In Fig 10, we see the effect of real-time immune rate on ℵ for classical and FF model. We see as we increase the immune rate, the number of infected nodes goes on decreasing in both models. As a comparison of both models, we see that no. of infected nodes is greater in FF model, which tells that in FF model nodes have stronger memory effect that enhances its sensitivity to network structure. Similarly Fig 11 shows that as the immune rate increases, no. of recovered nodes also increases in both models but in FF model this number is less than in classical model. It describes weak memory effect and decreased sensitivity in FF model.

**Fig 12:**
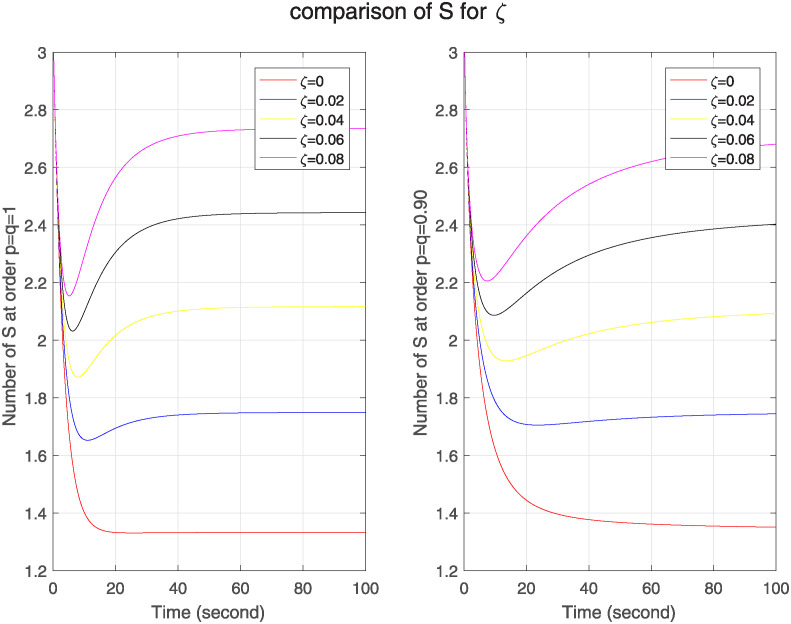
The effect of varying loss rate of immunity *ζ* on suspected nodes for classical and FF model at level 0.90.

Moreover, the recovered nodes lose their immunity after some time, so to see this impact we check the graphs of Δ, ℵ and Θ. We see in Fig 12 that in both models as the loss rate of immunity is increasing, no. of susceptible nodes is also increasing that describes weaker memory effect and higher sensitivity but in comparison FF model has stronger memory effect than classical one except at zero value.

**Fig 13:**
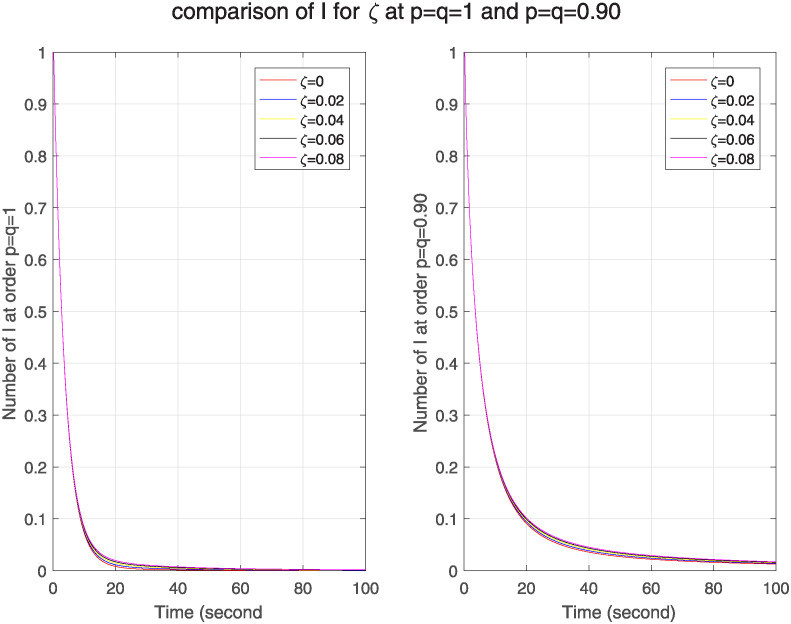
The effect of varying loss rate of immunity *ζ* on infected nodes for classical and FF model at level 0.90.

**Fig 14:**
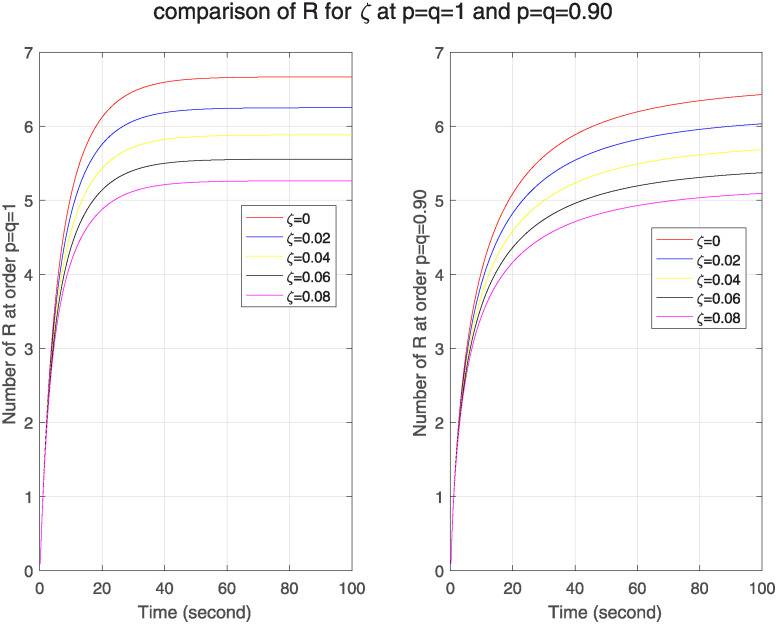
The effect of varying loss rate of immunity *ζ* on removed nodes for classical and FF model at level 0.90.

In Fig 13, we see the effect of loss rate of immunity on ℵ for both models. We see as loss rate of immunity increases, the number of infected nodes goes on decreasing in both models which indicates stronger memory effect. As a comparison of both models, no. of infected nodes is greater in FF model, which tells that in FF model nodes have weaker memory effect that decrease its resilience to network structure. In Fig 14 as loss rate of immunity increases, the no. of removed nodes is also increases, it indicates that immune response is effective and has strong memory effect. When we compare both models, we see that no of removed nodes is less in FF model. Hence, we say that theses nodes have weaker memory effect in FF model as compared to classical.

## 10 Conclusion

In this paper, a deterministic mathematical model on malware propagation has been discussed in the sense of fractal fractional derivative. At first stage the classical mathematical model given in [6] has been converted in fractal fractional model with power law kernel. Initially this model was examined theoretically. Conditions for existence (Leray Schauder criteria), uniqueness (Lipschitz property) and stability (Ulam-Hyers and Ulam-Hyers-Rassias theorems) of the fractal fractional model were examined using concepts of fixed point theory. Secondly, numerical scheme was developed and simulations were performed to verify the accuracy of theoretical results.

At second stage, fractal fractional model was examined under fractal dimensions and fractional orders separately. Then combined effect of fractal dimensions and fractional orders was discussed. We observed that at lower FF orders, the number of susceptible and infected nodes was higher. It demonstrates the sensitivity to external influences, resilience to adapt infection and strong memory effects. Under combined effect, we found out that removed nodes have higher containment of infection and persistence at lower level of FF orders.

At the next stage, we compared Δ, ℵ and Θ model for classical (FF order at one) and fractal fractional model for orders p=q=0.90. We examined the impact of different parameters such as initial infection rate, variable adjustment to sensitivity of infected nodes, immune rate of antivirus strategies and loss rate of immunity of recovered nodes of mathematical model [6] under p=q=1 and p=q=0.90.

Through the graphs we find out the effect of memory on different types of nodes in system. We explore sensitivity, convergence, and stability of susceptible, infected, and removed nodes under fractal fractional model. It will help us to predict about the vulnerabilities in computer systems. Antivirus strategies can be made by developing software that may help in containment and eradication of infection in the nodes by keeping an eye on the behavior of nodes. In classical form, this model gave a clear insight that by choosing appropriate variable infection rate, the prevalence of malware can be controlled. Our fractal fractional model agrees with it. Continuing this process, we investigated the impacts of other parameters too on malware model. Our findings may be helpful in installing antivirus software in cyber security practice by keeping in view the past behaviors of previous nodes. This model is suitable for malware like red worms, Nimda, Slammer worms, and Wittyworms etc. The malware which depends on variable infection rate and time delay factor, in that case our findings will help in developing antivirus strategies keeping in mind its cost factor.

This study has some limitations. Unavailability of sufficient real data causes hurdles to investigate the results. In future, we want to explore the behavior of mathematical model with respect to different kernels and different numerical schemes and by fractional and fractal derivatives of variable order so that more effective antivirus strategies may be developed to predict outbreaks for controlling malware propagation.

## Supporting information

S1 Dataset (DOCX)
