# Supplementary material for: Applying fractional calculus to malware spread: A fractal-based approach to threat analysis
Source: PLoS One. 2025 Jan 8;20(1):e0313914. doi: 10.1371/journal.pone.0313914 (PMC11709322; doi:10.1371/journal.pone.0313914)
Supplement: S1 Dataset — (DOCX) [file pone.0313914.s001.docx]

**Values of Parameters:**

Π = 0.5, θ = 0.8, ζ = 0.01, μ = 0.1, ν = 0.2, κ = 0.2, τ = 7.3, β = 0.02, α = 1 and some estimated initial conditions ∆(0) = 3, ℵ(0) = 1, Θ(0) = 0.1. Here

Π shows the susceptible rate of new nodes,

Θ shows the number of new nodes,

ζ is the loss rate of immunity of the recovered nodes,

μ is the replacement rate,

ν is the real time immune rate of antivirus strategies,

κ is the recovered rate of infected nodes,

τ is the change in time,

β_0_ is the initial infection rate,

α is used to adjust the infection rate sensitivity to ℵ.
